# Role of Elevated Ozone on Development and Metabolite Contents of Lemongrass [*Cymbopogon flexuosus* (Steud.) (Wats.)]

**DOI:** 10.3390/metabo13050597

**Published:** 2023-04-27

**Authors:** Parvati Madheshiya, Gereraj Sen Gupta, Ansuman Sahoo, Supriya Tiwari

**Affiliations:** Laboratory of Ecotoxicology, Centre of Advanced Studies, Department of Botany, Institute of Science, Banaras Hindu University, Varanasi 221005, India; parvati.madheshiya10@bhu.ac.in (P.M.);

**Keywords:** lemongrass, enzymatic antioxidant, biomass, medicinal properties

## Abstract

The present study was conducted to assess the effect of elevated ozone stress on the development and metabolite contents of lemongrass, a medicinal plant. The experimental plant was exposed to two elevated ozone concentrations (ambient + 15 ppb, and ambient + 30 ppb) using open-top chambers. Samplings were carried out at 45 and 90 days after transplantation (DAT), for the analysis of different characteristics, while the metabolite contents of leaves and essential oils were analyzed at 110 DAT. Both the doses of elevated ozone had notable negative effects on the carbon fixation efficiency of plants, resulting in a significant reduction in plant biomass. Enzymatic antioxidant activity increased during the second sampling, which suggests that the scavenging of reactive oxygen species was more prominent in lemongrass during the later developmental stage. The results of the present study showed a stimulated diversion of resources towards the phenylpropanoid pathway, which is made evident by the increase in the number and contents of metabolites in foliar extract and essential oils of plants grown at elevated ozone doses, as compared to ambient ozone. Elevated ozone not only upregulated the contents of medicinally important components of lemongrass, it also induced the formation of some pharmaceutically active bio compounds. On the basis of this study, it is expected that increasing ozone concentrations in near future will enhance the medicinal value of lemongrass. However, more experiments are required to validate these findings.

## 1. Introduction

Mean ground-level ozone (O_3_) concentration is continuously increasing due to accelerated development of several anthropogenic activities, including industrialization and vehicular emissions, over the past few decades [1,2]. Background average O_3_ concentrations have escalated from 20–30 ppb to 30–50 ppb and are expected to further increase up to 40–60% by the end of this century [3,4]. Anthropogenic activities have resulted in an almost three-fold increment in the emissions of oxides of nitrogen (NOx), volatile organic compounds (VOCs), and carbon monoxide (CO) [5]. This, coupled with the favourable meteorological conditions, has stepped-up the complex chemical reactions for the formation of this oxidative photochemical secondary air pollutant [6,7] in both developed and developing countries [8]. South Asian countries are continuously exposed to high O_3_ concentrations due to unchecked high emissions of O_3_ precursors and will continue to follow this same trend in the coming years [9,10,11]. However, simulation analyses have shown a significant reduction in O_3_ concentration in Europe and North America due to lower O_3_ precursor levels [12,13]. Several studies reported that suitable meteorological conditions have made China and India major O_3_ hotspots, with predicted increases of 13% and 20% in O_3_ precursors, respectively [14,15,16]. NOx levels in India showed an annual increasing trend of 0.9 ppb from 2010 to 2015 [8]. In India, the middle Indo-Gangetic Plains have seen high concentrations in the last few years, as made evident by a number of studies [17,18]. 

The pharmaceutical properties of medicinal plants are mainly attributed to their secondary metabolites (SMs) such as alkaloids, flavonoids, triterpenes, tannins, and phenolic acids [19,20]. Different medicinal plants are recognized as a source of natural free radicals scavenging compounds and are crucial in the chemoprevention of many diseases [21,22]. Owing to the presence of a substantial amount of SMs in medicinal plants have a number of pharmaceutical properties, such as anti-inflammatory, anticancer, antioxidative properties, which establish their use as herbal medicines [23]. Secondary metabolites are also known to play an important role in plant–environment interactions, coping with numerous biotic and abiotic stresses. It has been observed that environmental stress such as O_3_ pollution causes significant alterations in plants’ metabolic profiles [24,25,26]. Ground-level O_3_ generates oxidative stress in plants via the generation of reactive oxygen species, which disturbs normal cellular homeostasis, bringing about alterations in various morphological, physiological and biochemical processes [25,27,28]. The oxidative burst experienced by the plants under O_3_ exposure stimulates the antioxidant production and biosynthesis of SMs [29]. A number of SMs such as terpenoids, flavonoids, phenolics are observed to increase under elevated O_3_ [25,30]. Most O_3_-based studies have focused on the nutritional contents of agricultural crops and their bioactive phytochemical constituents are completely ignored. In the light of the recent COVID-19 wave(s), there has been an increasing demand in society for natural immunity boosters, and medicinal plants can successfully fill this void. As such, it becomes important to evaluate the qualitative and quantitative response of SMs of medicinal plants towards unfavourable environmental factors such as O_3_ stress. *Cymbopogon flexuosus* (Steud.) (Wats.) is an aromatic perennial C_4_ grass, grown in almost all tropical and subtropical countries and is an important source of bioactive secondary metabolites, commonly used in traditional medicines [31,32]. Lemongrass can be used for its analeptic, antioxidative, anti-inflammatory, and anticancer properties and is helpful in controlling blood sugar, cholesterol, and blood pressure. Citral, the major bioactive compound present in the essential oil of lemongrass, mainly comprises two stereoisomeric monoterpene aldehydes, geranial (C_10_H_16_O) and neral (C_10_H_16_O), exhibiting multitudinous therapeutic properties [31,33]. The present study was planned with the following objectives: (i) to evaluate the response of lemongrass to elevated O_3_ (as predicted in the coming years) by studying its morphological, biochemical and physiological characteristics, and (ii) to investigate the qualitative and quantitative alterations in the secondary metabolite profile of lemongrass upon exposure to elevated O_3_. We hypothesize that O_3_ stress initiates the upregulation of bioactive phytochemical compounds in lemongrass. We further aim to explore the mechanistic modifications and the compensatory influences on other plant functional characteristics, which facilitates the enhanced therapeutic importance of lemongrass under elevated O3 stress.

## 2. Materials and Methods

### 2.1. Study Site

The experiment was carried out between September 2021 and February 2022 in the Botanical Garden of Banaras Hindu University, a suburban area of Varanasi, Uttar Pradesh, located in the eastern Gangetic plains of the Indian subcontinent at 25°18 N, 82°01 E (76.19 m above mean sea level). The experimental site is characterized by alluvial, pale brown soil with a typical sandy loam texture comprising ~45% sand, 28% silt and 27% of clay, with pH ranging from 7.2 to 7.6 ± 0.2, which was measured using a soil pH meter (Eutech Cyber Scan pH 510, India). Total soil nitrogen and organic carbon contents were 0.21% and 1.44%, respectively. The meteorological parameters of the site were obtained from the Indian Meteorological Division (IMD), B.H.U., Varanasi. The study area experienced a mean monthly maximum temperature ranging from 31.68 ± 2.1 to 33.42 ± 1.25 °C and mean monthly minimum temperature between 8.42 ± 4.1 and 20.64 ± 1.14 °C, with a total rainfall of 855.20 mm that mostly occurred in September. Relative humidity varied from 84.5% to 90.93% during the experimental period. Daily mean sunshine duration ranged from 4.3 h to 11.2 h.

### 2.2. Plant Material

*Cymbopogon flexuosus* (Steud.) (Wats.) (Lemongrass), var. ‘Krishna’, a well-known medicinal and aromatic plant (MAPs), [34] was selected as the experimental material. *C*. *flexuosus* is a perennial C_4_ species widely in grown South Asian countries. Lemongrass is cultivated over an area of 16,000 ha throughout the world, generating around 1000 t of essential oil per year [35]. ‘Krishna’ is considered a superior variety as it has the potential to increase oil yield by up to three times compared to the other existing varieties [36]. Out of the top four ranked varieties of lemongrass, ‘Krishna’ is second for essential oil yield [9]. In India, the ‘Krishna’ variety can produce up to 300–369.00 q/ha fresh herb yield with 0.8–0.9% essential oil content, thus yielding 101.42 kg oil/ha containing 76–80% citral, an important medicinal component of lemongrass [37]. The lemongrass saplings were purchased from the Central Institute of Medicinal & Aromatic Plants, Lucknow (CSIR-CIMAP), Lucknow, India. The field was prepared following standard agronomic practices. The soil was prepared using recommended NPK doses for lemongrass (30:30:30) (National Horticulture Board, GOI) as basal dressing before planting saplings. 

### 2.3. Experimental Design

The experiment was performed in specially designed open-top chambers (OTCs), with 1.5 m diameter and 1.8 m height that were connected with a high-speed blower (168 ls^−1^) maintaining the near natural environment. The other structural and functional aspects of the chamber were followed as per Tiwari et al. [38]. The experiment consisted of three O_3_ doses; ambient (AO), ambient + 15 ppb (EO_1_), and ambient + 30 ppb (EO_2_). Elevated O_3_ was provided via O_3_ generators (A1G, Faraday, India), attached to the high-speed blowers of the OTCs. O_3_ treatment was started 25 days after transplantation (DAT) and carried out between 10.00 and 15.00 h. until maturity 

### 2.4. Ozone Monitoring

O_3_ concentrations were monitored for 8 h daily from 9.00 a.m. to 5.00 p.m. during the study period (Model APOA-370, HORIBA Ltd., Kyoto, Japan). O_3_ concentrations were measured via drawing air through a Teflon tube 0.35 cm in diameter, which was kept above the plant leaves inside the chamber. For each month of the experimental period, the AOT40 value was also calculated as per the formulae given by Mauzerall and Wang [39].

$$\mathrm{AOT}40=\sum_{i=1}^{n}\left[CO_{3}-40\right]$$

where, ‘*i*’ is the index, ‘*CO*_3_′ is the mean O_3_ concentration per hour ppb and ‘n’ designates the hours (in number) during which the O_3_ concentration was above 40 ppb/h.

### 2.5. Plant Sampling and Assessment of Plant Parameters

Leaf samples were randomly taken from OTCs with ambient and elevated O_3_ (EO_1_ and EO_2_) exposures for the estimation of different growth, biomass, physiological and biochemical parameters from each treatment after fumigation at 45 and 90 DAT (days after treatment). 

#### 2.5.1. Growth and Biomass Parameters

Triplicate plants with intact roots were randomly and carefully dug via digging a monolith of 10 × 10 × 25 cm^3^ from OTCs under ambient and elevated O_3_ exposures at 100 DAT (days after treatment). Several growth parameters such as root and shoot length, number of leaves, leaf area, root and shoot fresh weight and tillers were measured. Leaf area was measured using a portable leaf area meter (Model Li- 3100, Li-COR, Inc., Lincoln, NE, USA). For the estimation of dry weight, each plant was separated into the root, shoot and leaf and then kept in the oven (80 °C) until constant weights were achieved. Dry and fresh weight was determined, and the cumulative addition of all plant parts gave the total biomass as g plants^−1^. For the measurement of foliar injury percentage (FIP), triplicate plants were collected at 100 DAT from all treatment and the total area of leaves was estimated for all plants, with or without symptoms measured using a portable leaf area meter (LI 3000, LICOR Inc., USA). According to Mishra et al. [40], FIP was calculated using the following formula:

$$\mathrm{FIP}=\frac{\mathrm{Total}\;\mathrm{leaf}\;\mathrm{area}\;\mathrm{injured}\;\times \;100}{\mathrm{Total}\;\mathrm{leaf}\;\mathrm{area}}$$

#### 2.5.2. Biochemical Assays

##### Hydrogen Peroxide (H_2_O_2_), Superoxide Radicals (O_2_^o−^) and MDA Content

H_2_O_2_ content was estimated using the method given by Alexieva et al. [41]. Leaf samples homogenised with trichloroacetic acid (TCA) were treated with potassium phosphate buffer and potassium iodide solution sequentially. The absorbance of the reaction mixture was taken at 390 nm using a double-beam spectrophotometer (model 2203, Systronics, India). For estimation of O_2_^o−^, the method described by Elstner and Heupel [42] was used, with a few modifications. Lipid peroxidation was estimated in fresh leaves tissue in terms of malondialdehyde (MDA) content as described by the methodology of Heath and Packer [43]. 

##### Antioxidants and Metabolites

Enzymatic antioxidants activities, such as superoxide dismutase (SOD), glutathione reductase (GR), ascorbate peroxidase (APX) and catalase (CAT) enzymes, were evaluated as described by Fridovich [44], Anderson [45], Nakano and Asada [46] and Aebi [47], respectively. Non-enzymatic antioxidants, such as phenols [48], flavonoids [49], anthocyanin [50], proline [51], alkaloids [52], and ascorbic acid (AsA) [53] were quantified using the respective protocols. 

##### ROS Scavenging Activity

For free-radical scavenging activities, such as 2,2-diphenyl-1-picrylhydrazyl (DPPH), 2,2′-azino-bis (3-ethylbenzothiazoline-6-sulfonic acid) diammonium salt (ABTS) and ferric reducing antioxidant power (FRAP), the radical scavenging assay followed the protocols of Noreen et al. [54], Khatua et al. [55] and Clarke et al. [56], respectively. 

#### 2.5.3. Measurement of Gas Exchange and Photochemical Efficiency

On three plants from each OTC, measurements in triplicate were carried out for gas exchange parameters, such as internal CO_2_ (*iCO*_2_), stomatal conductance (*gs*), transpiration rate (E), net photosynthesis rate (*Ps*), and water usage efficiency (WUE), at 65 DAT using Ciras-3 Portable Photosynthesis System (PP Systems, Haverhill, Amesbury, MA, USA) with 25 mm 97 mm size PLC3 universal leaf cuvette. The measured physiological characteristics were randomly studied on the third fully expanded leaf from each treatment plant, oriented to normal irradiation during cloud-free days. The portable photosynthetic system took measurements between 08:00 h and 10:00 h. During the measurements, photosynthetically active radiation (PAR) ranged between 1100 and 1200 μmol m^−2^s^−1^. The system was calibrated using a known CO_2_ source (509 ppm). Gas measurements were made at a constant flow rate (500 μmol s^−1^). Maximum photochemical efficiency of PSII (Fv/Fm) was measured after the initial 30 min dark-adapted leaves [57] using PAM-2500 WALZ (PAMWin-3 software), Germany.

#### 2.5.4. Photosynthetic Pigments

For pigment contents (total chlorophyll and carotenoids) determination, 0.5 g of fresh leaves homogenized in 20 mL of 80% acetone were centrifuged for 15 min at 4032× *g*. Optical densities were taken at 645 nm and 663 nm wavelengths for chlorophyll a and b contents, respectively, and at 480 and 510 nm for carotenoid content using a double-beam spectrophotometer (Model-2203, Systronics, India). Chlorophyll and carotenoid content were estimated using the formulae developed by Takshak and Agrawal [58].

#### 2.5.5. Isolation of Essential Oil

Fresh shoot samples of lemongrass were taken from OTCs under ambient and elevated O_3_ exposures, including control at 110 DAT following the formulae of Takshak and Agrawal [58] with some modifications. Triplicate samples were washed thoroughly with distilled water, chopped finely and processed via hydro distillation for four hours in a Clevenger-type apparatus [59]. After being extracted, the essential oil was separated, dried over anhydrous sodium sulphate, and refrigerated at 4 °C until analysis. The volume of essential oil obtained was estimated, and the percentage of oil content was measured using the weight of fresh shoot samples. The samples were further subjected to chemical characterization of essential oil analysed using GC–MS (gas chromatography–mass spectroscopy) (QP-2010, Shimadzu, Japan).

#### 2.5.6. Extraction, Analysis, and Identification of Different Metabolites of Leaf

At 110 DAT, randomly selected leaves collected from OTCs under ambient and elevated O_3_ exposures, including control samples, were cut into small pieces and dried at room temperature (25–30 °C) in the shade. An electric grinder was utilized to crush the dried leaves to powder, and the leaves were soaked in 90% HPLC-grade methanol for 72 h with intermittent shaking [60] then filtered. Next, 10 mL supernatant was treated with 20 mL 0.5 N H_2_SO_4_. To make the solution alkaline, a few drops of NH_4_OH and further chloroform added; the solution was then properly mixed and kept at room temperature for 24 h for incubation. Two layers formed, among which the upper layer was discarded and the lower layer containing the majority of the phytoconstituents was concentrated to 2 mL through a cold rotavapor and subjected to GC–MS analysis [25].

### 2.6. GC–MS Conditions and Specifications

The leaf extracts and essential oil samples of all treatments were prepared and analyzed with the help of GC–MS (QP-2010, Shimadzu, Japan) with a capillary (30 m× 0.25 mm i.d. × 0.25 μm film thickness) column (Rtx-5Ms). Then, 1.0 μL leaves extracts or essential oil samples were injected into the system at 260 °C with helium gas as a carrier at a flow rate of 1.21 mL min^−1^. Initially, the temperature of the oven was set at 500 °C for 2 min, followed by an increment in temperature to 210 °C at a rate of 3 °C min^−1^ and a holding time of 2 min. This was again increased to 2800 °C at 80 °C min^−1^ and maintaining for 9 min, a pattern that remained until the end of the running program. The pressure of 73.3 kPa, total flow of 16.3 mL min^−1^, linear velocity of 3.0 mL min^−1^, and a split ratio of 10:0 was all maintained throughout GC. The operating values were as follows: ion source temperature, 230 °C; interference temperature, 270 °C; solvent cut time, 3.5 min; scan speed, 333 amu s^−1^; MS running time, 60.6 min; and threshold, 1000. The metabolites of the leaves extracts and essential oil samples were identified by comparing their retention indices and mass spectra fragmentation patterns with those compounds reported within libraries (NIST14, NIST14s, WILEY8, FFNSC, and SZTERP) using the GC–MS data system.

### 2.7. Identification of Different Metabolites of Leaf Extract and Essential Oil

The database of the National Institute of Standards and Technology, which contains more than 62,000 patterns, was used to identify the extracted metabolites. The spectra of unknown components were compared to known components contained in the WILEY and NIST libraries (WILEY8.LIB and NIST14.LIB), which provide the molecular weight, chemical structure, and molecular formula of the plant’s constituent parts.

### 2.8. Statistical Analysis

All statistical analyses were performed using the SPSS statistical package (SPSS Inc., version 16.0.0). Initially, the Shapiro–Wilk test was used to determine the normality of each dataset. The distribution was considered normal as, in every case, the *p* values were above 0.05 (level of significance). Further principal component analysis (PCA), regression method, different graphical representation and one-way ANOVA (analysis of variance) were used to test the effect of O_3_ on the physiological, biochemical, morphological and yield characteristics of medicinal plants. After subjecting to a one-way ANOVA test on physiological, biochemical, and morphological parameters, Duncan’s multiple range tests were performed as post hoc tests for various measurements. Each ANOVA was used to check the assumption of homoscedasticity. Sigma Plot 12.5 and GraphPad (Version 8.02) were used to plot the graphs and heatmaps. The percentage changes in all parameters were calculated with respect to control, i.e., AO. The PCA was performed using the correlation matrix, varimax rotation and regression method. All statistical analyses were performed using IBM SPSS/PC+ (ver. 25.0, x64).

## 3. Result

### 3.1. Ozone Monitoring and AOT 40

**Figure 1:**
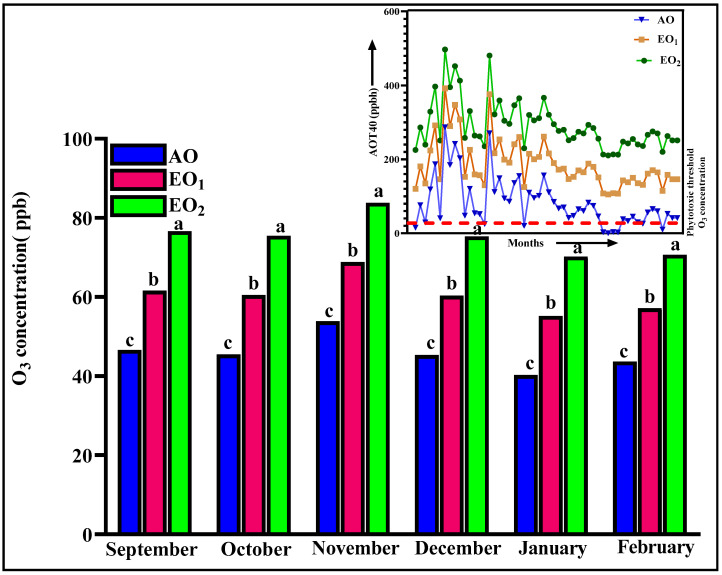
Daily 8 h ambient and elevated O_3_ concentration and AOT40 during the experimental period at the experimental site. Bars represent mean values. Bars showing different letters indicate significant differences according to Duncan’s test at *p* < 0.05.

The ambient mean O_3_ concentration throughout the experimental period was found to be 46.69 ± 2.1 ppb at the experimental site, which is above the phytotoxic threshold level. The maximum observed ambient O_3_ concentration was 53.85 ± 1.5 ppb and the minimum was 38.22 ± 1.3 ppb (Figure 1). The AOT40 value calculated through daily mean 8 h O_3_ concentration during the experimental period (September to February) was 3606.43 ppb/h. In EO_1_ and EO_2_ exposure, mean 8 h O_3_ concentrations throughout the experimental period were observed to be 61.49 ± 1.5 ppb and 76.30 ± 3.4 ppb and AOT 40 values for these concentrations were observed to be 11,586.43 ppm/h and 19,566.43 ppm/h, respectively (Figure 1). The maximum value of AOT40 recorded in December was 6446.53 ppm/month. 

### 3.2. Gas Exchange and Chlorophyll Fluorescence

**Figure 2:**
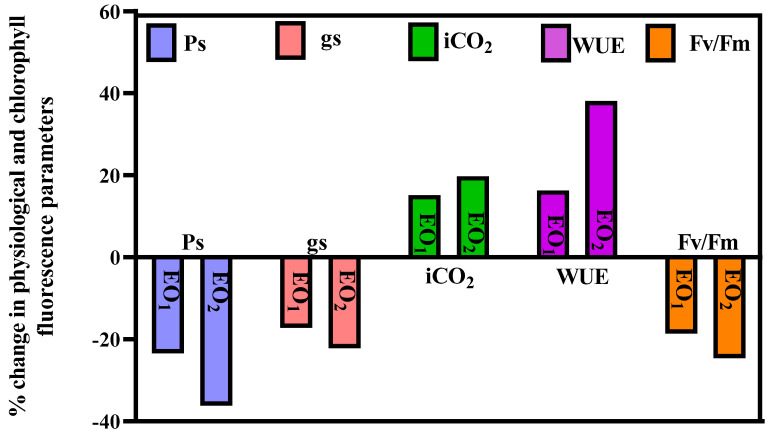
Percent change in different physiological parameters of lemongrass grown at elevated ozone concentrations (EO_1_ and EO_2_) at 65 days after transplantation (DAT) (*Ps* = rate of photosynthesis; *gs* = stomatal conductance; *iCO*_2_ = internal CO_2_; WUE = water use efficiency; Fv/Fm = chlorophyll fluorescence).

Significant variations of (−) 23.42, (−) 17.18, (+) 15.16, (+) 16.30 and (−) 18.63% were recorded for *Ps*, *gs, iCO*_2_, WUE and Fv/Fm, respectively, during EO_1_ treatment at 65 DAT (Figure 2). During EO_2_ treatment, *Ps, gs, iCO_2_*, WUE and Fv/Fm showed significant variations of (−)36.15, (−) 22.19, (+) 19.73, (+) 38.12 and (−) 24.63%, respectively (Figure 2).

### 3.3. Photosynthetic Pigments 

**Figure 3:**
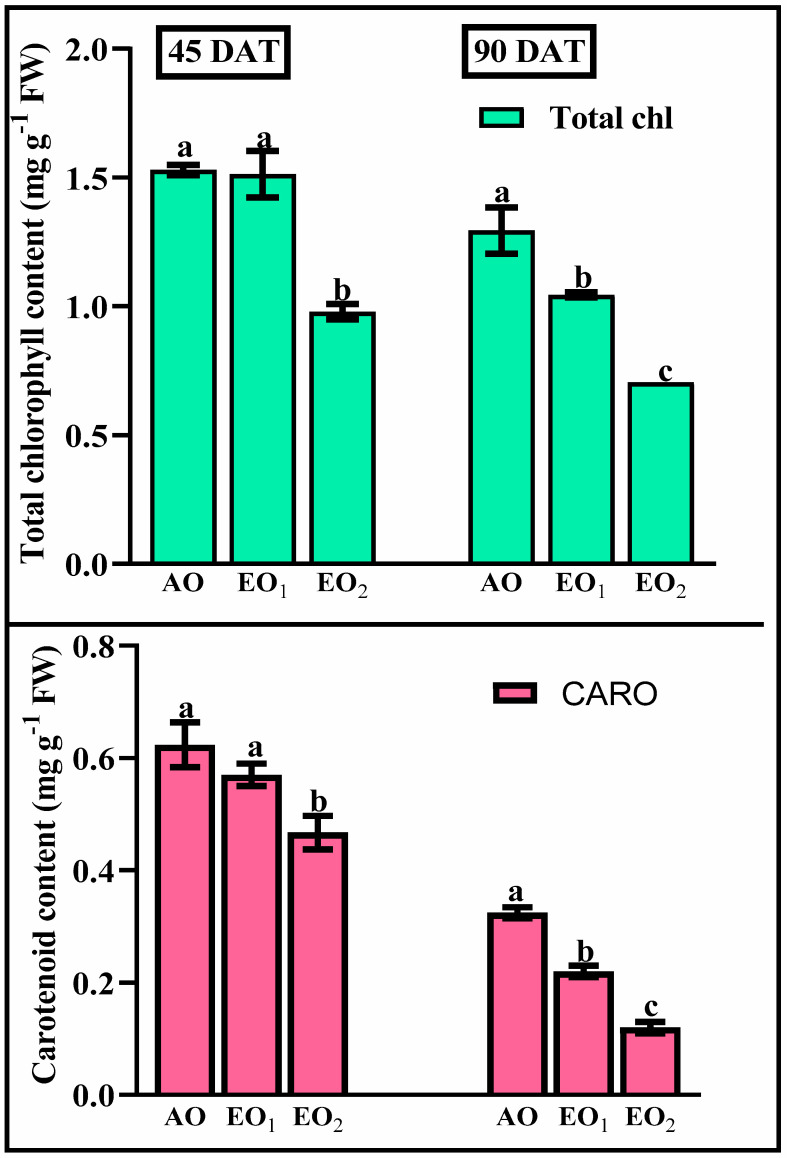
Effect of ambient (AO) and elevated O_3_ (EO_1_ and EO_2_) on the responses of photosynthetic pigments of lemongrass at 45 and 90 days after transplantation (DAT). Bars represent mean ± SE. Bars with different letters in the same group show significant variation at *p* < 0.05.

Total leaf chlorophyll and carotenoid content decreased by 1.06 and 8.59% during EO_1_ treatment and by 35.97 and 25.08% during EO_2_ treatment, respectively, compared to the control (*p* < 0.05) at 45 DAT (Figure 3). At 90 DAT, reductions of 45.62 and 62.21% during EO_1_ treatment and 19.45 and 30.73% during EO_2_ treatment, were observed, as compared to the control, respectively (*p* < 0.05).

### 3.4. Biochemical Assays

#### 3.4.1. Hydrogen Peroxide (H_2_O_2_), Superoxide Radicals (O_2_^o−^) and MDA Contents

**Figure 4:**
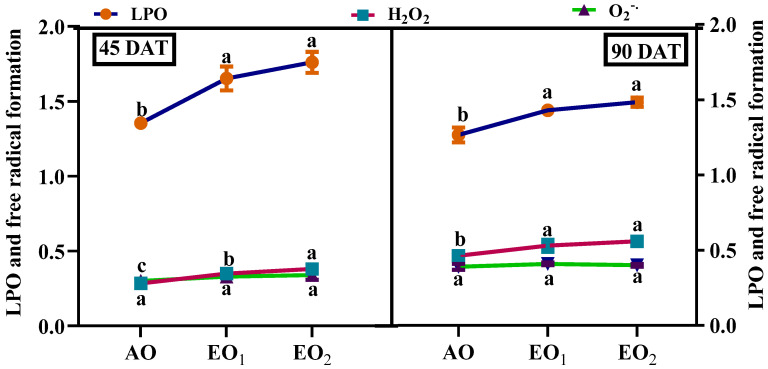
Effect of ambient (AO) and elevated O_3_ (EO_1_ and EO_2_) on the H_2_O_2_ and O_2_^−^ production and MDA content of lemongrass at 45 and 90 days after transplantation (DAT). Dots represent mean ± SE. Dots with different letters in the same group show significant variations at *p* < 0.05.

Significant increments in ROS and MDA contents were observed at elevated O_3_ concentrations during both the sampling stages. O_2_^o−^ contents significantly increased by 10.32 and 13.76%, respectively during EO_1_ and EO_2_ treatments compared to the control at 45 DAT (Figure 4). At 90 DAT, O_2_^o−^ contents did not show any significant variations. H_2_O_2_ contents significantly increased by 25.72 and 37.20% at 45 DAT and by 17.31 and 22.43% at 90 DAT during EO_1_ and EO_2_ treatments, respectively (Figure 4). MDA contents significantly increased by 22.37 and 30.16% at 45 DAT and by 13.17 and 17.16% at 90 DAT during EO_1_ and EO_2_ treatments, respectively (Figure 4). 

#### 3.4.2. Antioxidants and Metabolites

**Figure 5:**
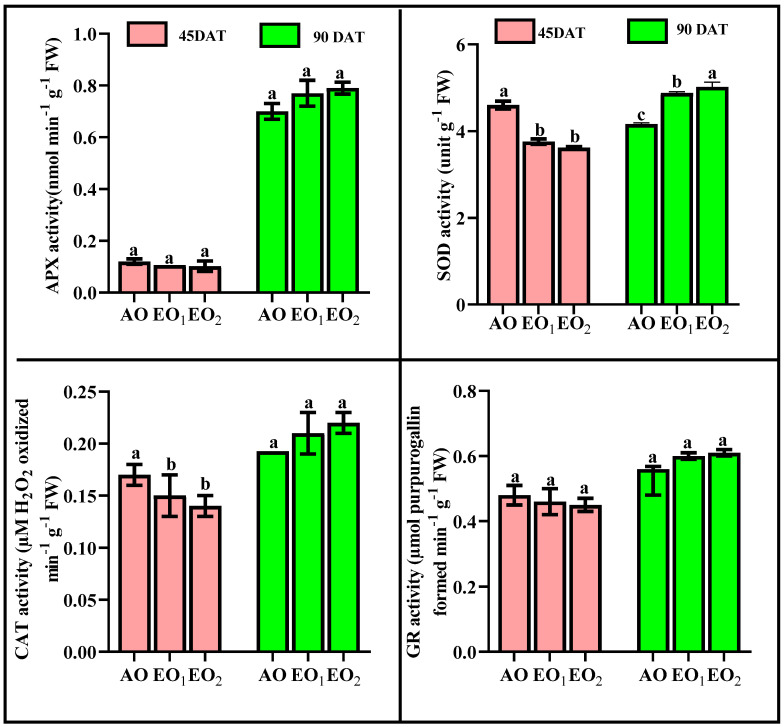
Effect of ambient (AO) and elevated O_3_ (EO_1_ and EO_2_) on the responses of enzymatic antioxidants of lemongrass at 45 and 90 days after transplantation (DAT). Bars represent mean ± SE. Bars with different letters in the same group show significant variation at *p* < 0.05. (APX = ascorbate peroxidase; SOD = superoxide dismutase; CAT = catalase; GR = glutathione reductase).

**Figure 6:**
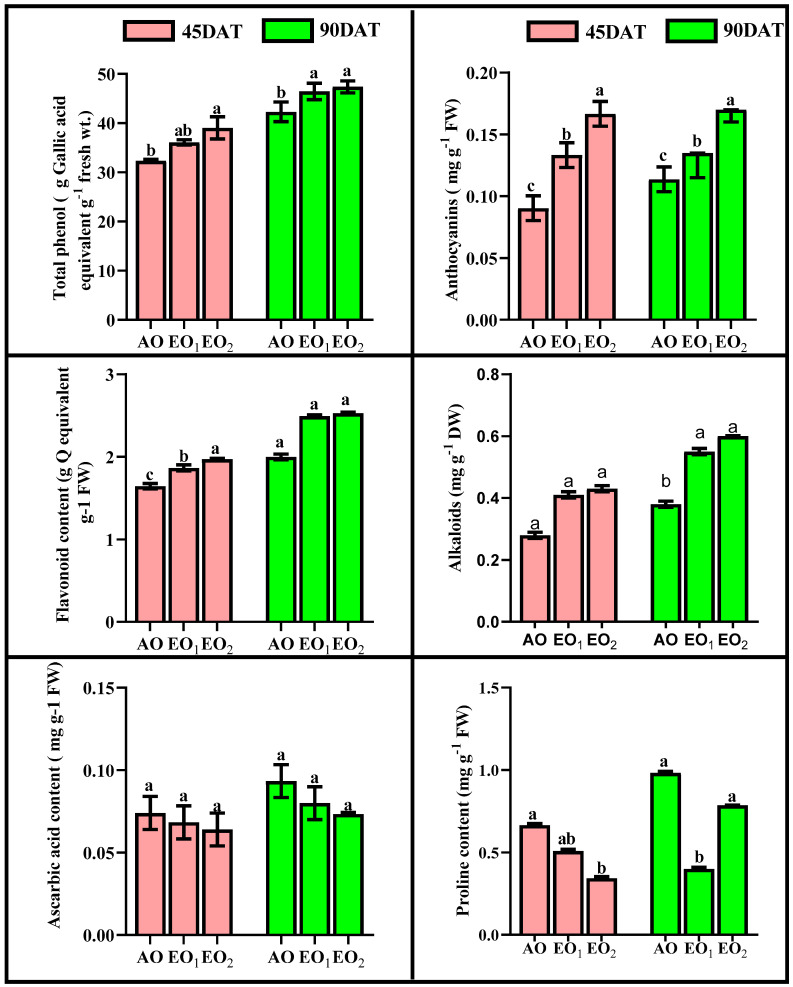
Effect of ambient (AO) and elevated O_3_ (EO_1_ and EO_2_) on the responses of selected metabolites of lemongrass at 45 and 90 days after transplantation (DAT). Bars represent mean± SE. Bars with different letters in the same group show significant variation at *p* < 0.05.

SOD activity significantly decreased by 18.30 and 21.36% at 45 DAT, while SOD activity significantly increased by 17.93 and 20.76% at 90 DAT during EO_1_ and EO_2_ treatments, respectively (Figure 5). APX activity significantly decreased by 12.38 and 15.41% at 45 DAT, while it significantly increased by 11.07 and 13.61% at 90 DAT during EO_1_ and EO_2_ treatments, respectively, as compared to the control (Figure 5). CAT activity also significantly decreased by 11.01 and 13.22% at 45 DAT, while it significantly increased by 12.81 and 15.78% at 90 DAT during EO_1_ and EO_2_ treatments, respectively, as compared to the control (Figure 5). At 90 DAT, GR activity significantly varied during EO_2_ treatment compared to the control (Figure 5). Phenol content increased by 11.64 and 20.78% at 45 DAT and by 9.79 and 12.0% at 90 DAT. Flavonoid contents showed significant increments of 13.48 and 19.86% at 45 DAT and by 24.5 and 26.0% at 90 DAT during EO_1_ and EO_2_ treatments, respectively, as compared with the control (Figure 6). At 90 DAT, AsA contents were significantly reduced by 11.11 and 14.28% in the EO_1_ and EO_2_ treatments, respectively, as compared with the control (Figure 6). Similarly, proline decreased by 23.54 and 48.41% at 45 DAT and by 59.31 and 20.06% at 90 DAT during EO_1_ and EO_2_ treatments, respectively, as compared with the control (Figure 6). Anthocyanins significantly increased by 47.6 and 84.5% at 45 DAT and 18.77 and 49.56% at 90 DAT during EO_1_ and EO_2_ treatments, respectively, as compared with the control. Similarly, alkaloid contents significantly increased by 46.43 and 53.57% at 45 DAT and 44.77 and 57.89% at 90 DAT during EO_1_ and EO_2_ treatments, respectively, as compared with the control (Figure 6).

#### 3.4.3. ROS Scavenging Potential

**Figure 7:**
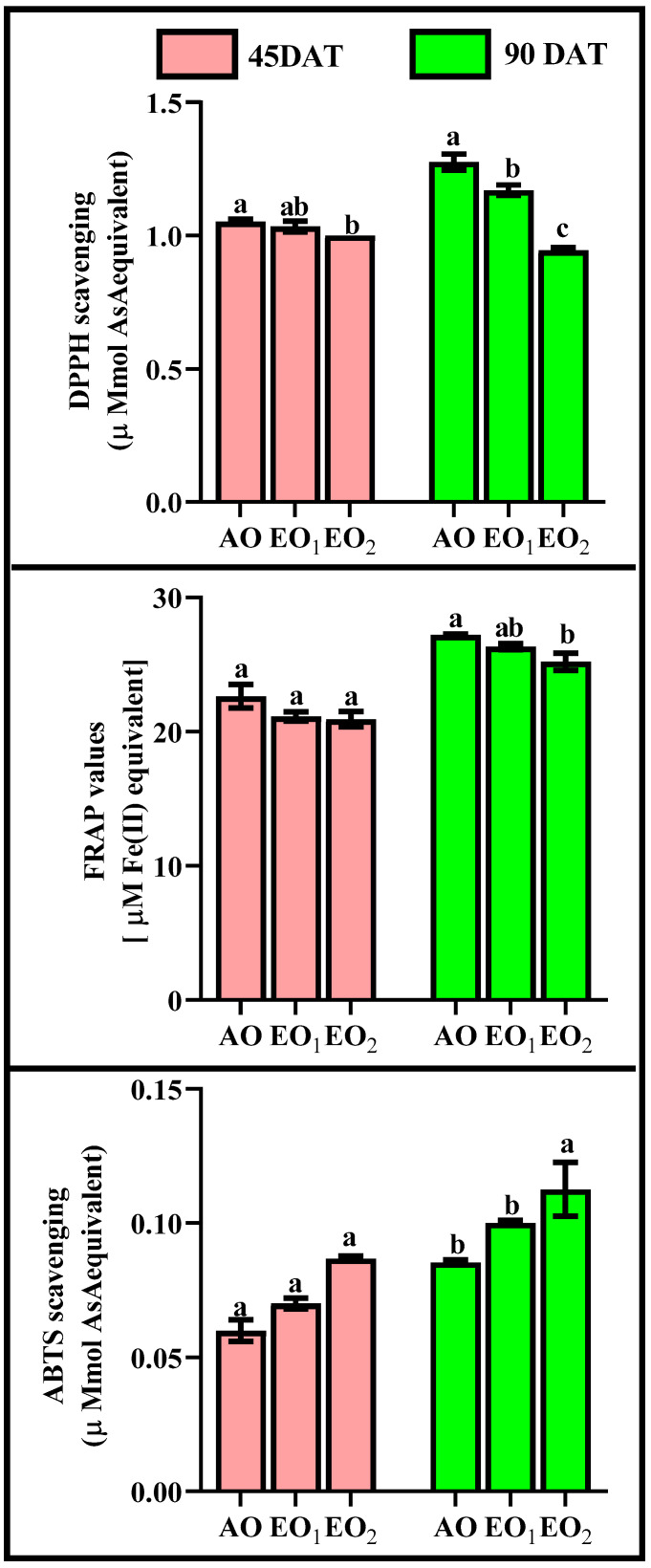
Effect of ambient (AO) and elevated O_3_ (EO_1_ and EO_2_) on the ROS scavenging potential measured in terms of DPPH, FRAP and ABTS of lemongrass at 45 and 90 days after transplantation (DAT). Bars represent mean ± SE. Bars with different letters in the same group show significant variations at *p* < 0.05.

DPPH significantly declined by 1.94 and 4.76% at 45 DAT and 8.59 and 26.56% at 90 DAT during EO_1_ and EO_2_ treatments, respectively, as compared with the control (Figure 7). FRAP also significantly declined by 6.69 and 7.60% at 45 DAT and 3.22 and 7.38% at 90 DAT, whereas ABTS significantly increased by 16.67 and 44.44% at 45 DAT and 17.29 and 32.00% at 90 DAT during EO_1_ and EO_2_ treatments, respectively, as compared with the control (Figure 7).

### 3.5. Morphological Parameters

**Figure 8:**
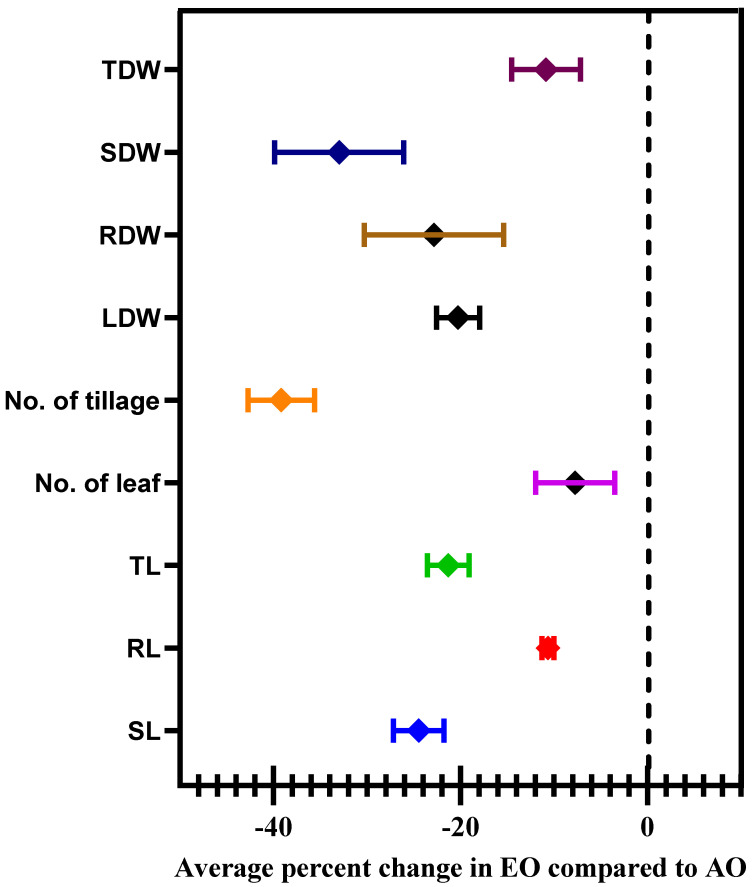
Average percentage change in selected growth and morphological characteristics of lemongrass at 100 days after transplantation (DAT) grown at elevated ozone concentrations (EO_1_ and EO_2_). Symbols represent the mean of % change, and error bars are the 95% confidence interval.

**Figure 9:**
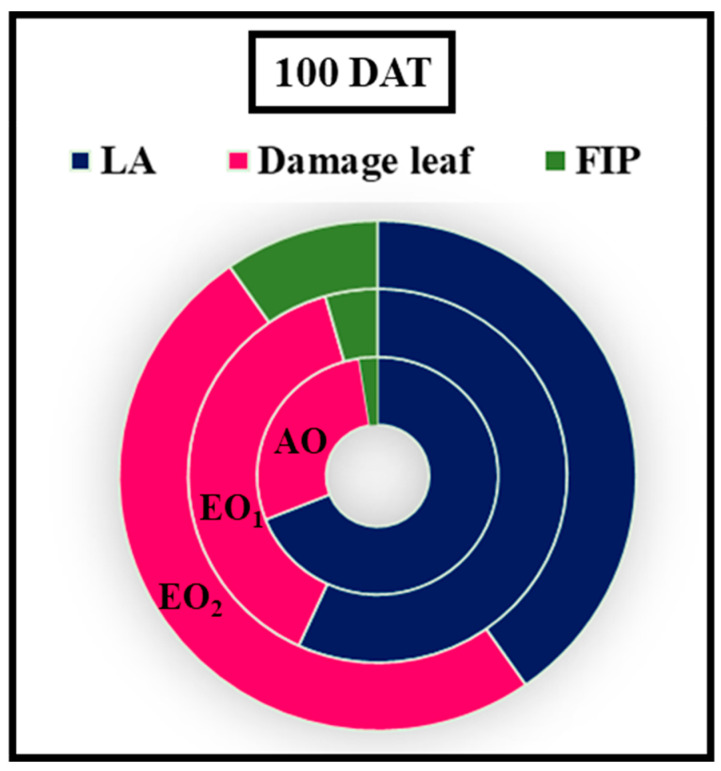
Foliar injury percent (FIP) and leaf area of lemongrass at 100 days after transplantation (DAT) grown at elevated ozone concentrations (EO_1_ and EO_2_).

At 100 DAT, the shoot length (SL) and root length (RL) of the plants were significantly decreased by 21.76, 26.14% and 9.07 and 11.11% due to EO_1_ and EO_2_ treatments, respectively, as compared to the control (Figure 8). At 100 DAT, number of leaves, number of tillages, and leaf area also showed significant reductions of 1.18 and 27.27%, respectively, during EO_1_ treatment, and 4.71 and 36.36%, respectively, during EO_2_ treatment, as compared to control (Figure 8). At 100 DAT, leaf area significantly reduced by 25.17 and 55.50% during EO_1_ and EO_2_ treatments, respectively, as compared with control (Figure 9). At 100 DAT, the estimated FIP values in mature leaves caused by EO_1_ and EO_2_ exposures were 68.09 and 124.12%, respectively, while 41.38% leaf injury was observed in plants grown under AO treatment (Figure 9). Total plant biomass significantly decreased by 7.11 and 36.52% at 100 DAT during EO_1_ and EO_2_ treatments, respectively, as compared with control (Figure 8).

### 3.6. Effects on Metabolite Constituents in Leaves Extract and Essential Oil

**Figure 10:**
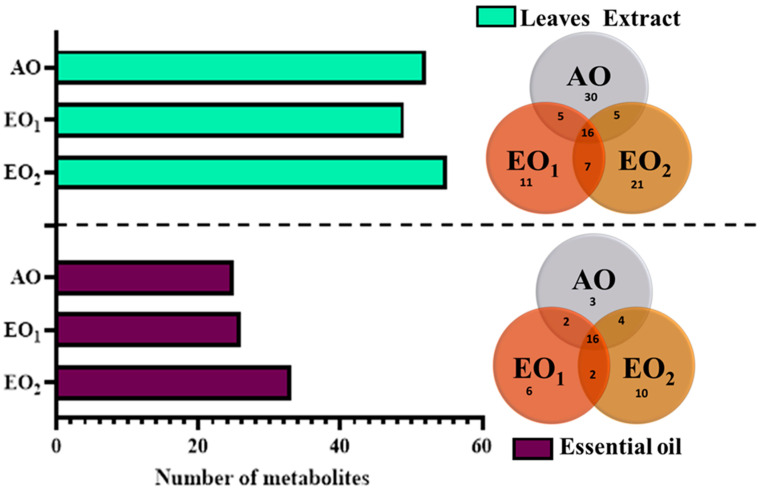
Number of metabolites observed via GC–MS analysis in ambient (AO) and elevated O_3_ (EO_1_ and EO_2_) in lemongrass at 110 days after transplantation (DAT). The Venn diagram shows the number of common and uncommon metabolites at different treatments.

**Figure 11:**
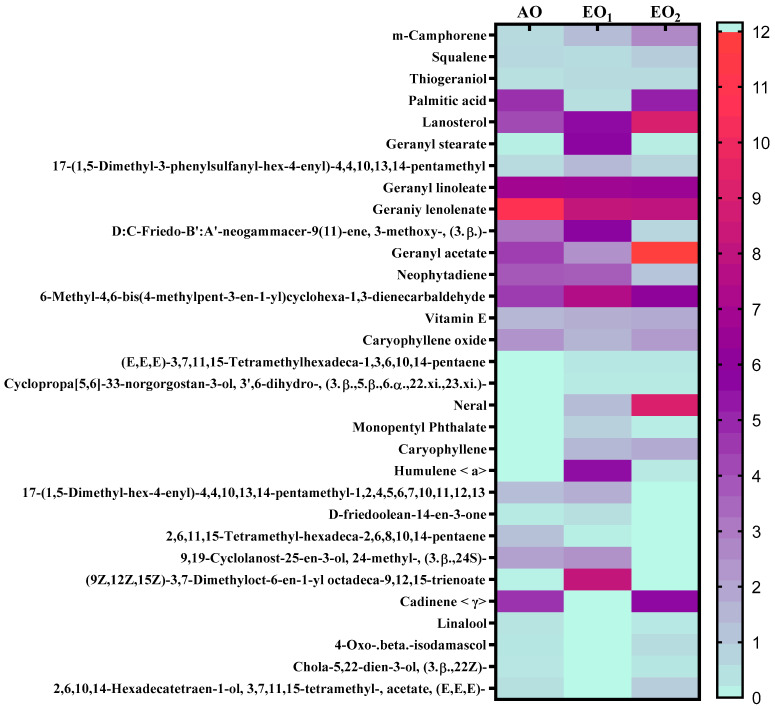
Heatmap showing variations in the percentage retention peak area in the contents of common metabolites observed in leaf extracts of lemongrass via GC–MS analysis, 110 days after transplantation (DAT) at ambient (AO) and elevated O_3_ (EO_1_ and EO_2_) treatments. Red shows the higher percentage retention peak area, purple represents medium percentage retention peak area, and light sea green represents the lower percentage retention peak area. Legend numbers represent the gradients of the retention peak area.

**Figure 12:**
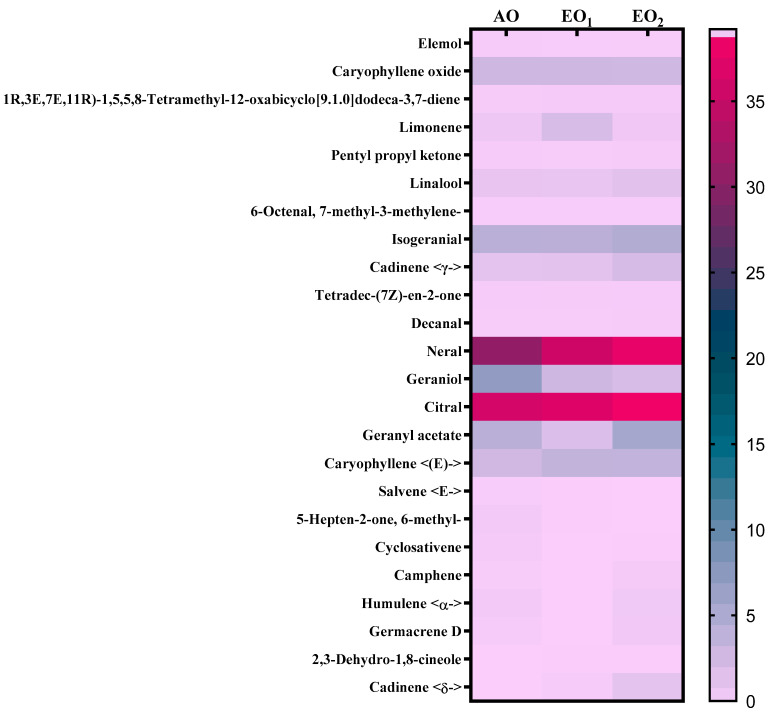
Heatmap showing the variations in percentage retention peak area in the contents of common metabolites observed in the essential oil of lemongrass via GC–MS analysis at 110 days after transplantation (DAT) at ambient (AO) and elevated O_3_ (EO_1_ and EO_2_) treatments. Pink shows the higher percentage retention peak area, teal blue represents a medium percentage retention peak area and purple represents a lower percentage retention peak area. Legend numbers represent the gradient of retention peak area.

Under EO_1_ and EO_2_ exposure, metabolites of both the leaf extract and essential oil of the test plants showed considerable variations as control (AO) leaf extract and essential oil. At 110 DAT, 54, 41 and 53 metabolites in leaf extracts, and 25, 26 and 33 metabolites were present in essential oil under AO, EO_1_ and EO_2_, respectively, (Figure 10). The variations in the composition of some pharmacologically important compounds are shown in Figure 11 and Figure 12 (Supplementary Tables S1 and S2). In AO leaf extract, out of 54 metabolites—2,6-octadien-1-ol, 3,7-dimethyl-, (E) (3.59%), geranyl acetate (4.62%), naphthalene (4.89%), 1,2,3,4,4a,5,6,8a-octahydro-7-methyl-4-methylene-1-(1-methylethyl)-, (1. α),neophytadiene (3.9%), hexane, 1-bromo-6-chloro-(3.05%), palmitic acid (4.94%), geranyl linolenate (10.87%), geranyl linoleate (6.99%), lanosterol (4.33%), d:c-friedo-b’:a’-neogammacer-9(11)-ene, and 3-methoxy-, (3. β.) (3.37%)—some had a maximum % concentration (Figure 11). On the other hand, some metabolites—neophytadiene (3.38%), glutaric acid, myrtenyl 3-methylbut-2-en-1-yl ester (4.43%), 6-Methyl-4,6-bis(4-methylpent-3-en-1-yl)cyclohexa-1,3-dienecarbaldehyde (7.63%), trans-p-mentha-2,8-dienol (3.87%), geranyl linoleate (6.83%), geranyl linolenate (8.33%) lanosterol (5.73%), and D:C-Friedo-B’:A’-neogammacer-9(11)-ene, 3-methoxy-, (3.b β.) (5.97%)—showed higher % concentration during EO_1_ exposure (Figure 11, Supplementary Table S1). Out of 53 metabolites, 13 metabolites—β-Citral (11.71%), geranyl acetate, (12.16%), 2,6 octadien-1-ol,3,7-dimethyl-, acetate (9.14%), Cadinene < γ> (5.78%), 6-methyl-4,6-bis(4-methylpent-3-en-1-yl)cyclohexa-1,3-dienecarbaldehyde (6.27%), palmitic acid(5.31%), geranyl linoleate (6.75%), geranyl linolenate (8.18%), and lanosterol (9.14%)—showed higher% concentrations in EO_2_ exposure (Figure 11, Supplementary Table S1). In the case of essential oil, citral is the principal active compound and its concentration determines the quality of the oil. A remarkably high percentage of neral and geranial was found in the oil due to AO, EO_1_ and EO_2_ exposure. The increases in the concentration (%) of Geranial (C_10_H_16_O) contents by 36.7, 37.66, and 39.21% and neral (C_10_H_16_O) contents by 31.33, 36.23 and 38.42% were caused by AO, EO_1_ and EO_2_ exposure, respectively (Figure 12, Supplementary Table S2). Some of the metabolites such as isogeranial, geranyl acetate, geranyl linalool decreased during EO_1_ treatment and increased during EO_2_ exposure compared to AO exposure. On the other hand, limonene and tetradec-(7Z)-en-2-one levels increased during EO_1_ treatment but decreased during EO_2_ treatment, and neral, decanal, caryophyllene <(E)-> and cadinene < γ-> increased. Some important metabolites such as caryophyllene oxide and geraniol decreased during O_3_ exposure in essential oil. Some metabolites were detected only under AO conditions—(2,2-dimethylocta-3,4-dienal, citronellal and cubebol), EO_1_ (bisabolene < (E)- γ- >, α-ylangene, humulene < α>, germacrene D, bisabolene < (E)- γ- > and cuparene) and EO_2_ (cubebanol, germacrene a, bisabolene < (Z)-, γ->, cadina-1,4-diene <trans->, and epicubenol exposure. Similarly, 2,3-dehydro-1,8-cineole and cadinene < δ> increased in both EO_1_ and EO_2_ exposure (Figure 12, Supplementary Table S2). Details of variations in the contents of different metabolites during different O_3_ treatments are shown in the supplementary data (Supplementary Tables S1 and S2), along with the IUPAC names and the chemical structures of the different metabolites (Supplementary Table S3).

## 4. Discussion

**Figure 13:**
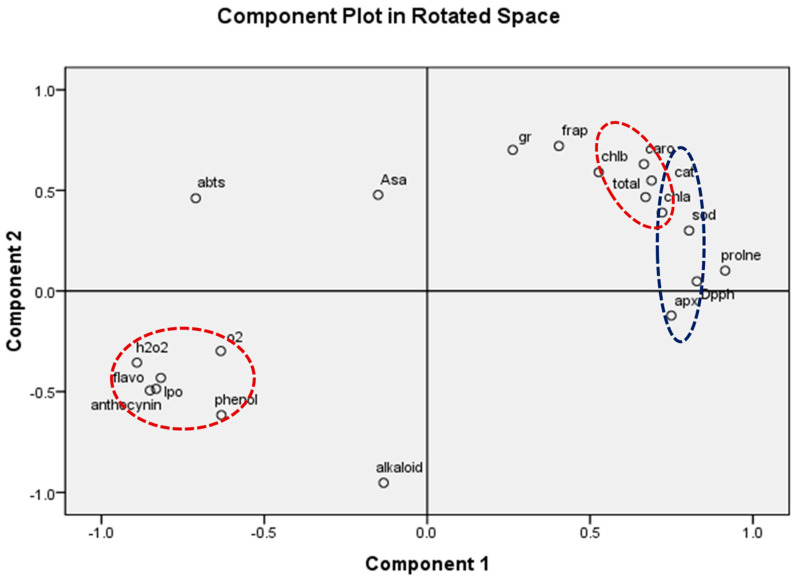
Principal component analysis (PCA) showing the effect of ambient (AO) and elevated O_3_ (EO_1_ and EO_2_) on considered biochemical parameters of lemongrass. The parameters studied are total Chl (total chlorophyll), Caro (carotenoids), AsA (ascorbic acid), phenol, flavonoid, DPPH, ABTS, FRAP, alkaloid, proline, anthocyanin, APX (ascorbate peroxidase), GR (glutathione reductase), SOD (superoxide dismutase), catalase (CAT), lipid peroxidation (LPO), H_2_O_2_ (hydrogen peroxide) and SOR (superoxide radical) Red dotted circles showed the synchronization of H_2_O, SOR, LPO, anthocyanin phenol, flavonoid, Caro, and total Chl. Similarly blue dotted circles showed the synchronization of APX, DPPH, CAT and SOD parameters.

The present experimental site is well marked by a high concentration of O_3_ as evidenced by a number of previous studies [1,61,62,63]. The present study reported 8 h average O_3_ concentration of 46.69 ± 2.1 ppb, high enough to cause noticeable damage to the plants. In the present experiment, the negative effects of O_3_ are clearly demonstrated in the biochemical and physiological responses of lemongrass. The first indicator of O_3_-induced biochemical damage is the increased concentration of malonaldehyde (MDA), which acts as a marker of oxidative stress in plants [4,27,58,62]. Increased malonaldehyde content in O_3_-exposed plants is a result of lipid peroxidation, which suggests a disruption in plasma membrane permeability leading to an unbalance in disrupted cellular homeostasis [64]. Enhanced lipid peroxidation is attributed to the stimulated production of reactive oxygen species (ROS) such as O_2_^−0^ and H_2_O_2_ [65] at high O_3_ concentrations. It was observed that, in the present study, the magnitude of lipid peroxidation and ROS production increased with increasing O_3_ concentration, signifying higher oxidative stress at higher O_3_ (AO < EO_1_ < EO_2_) concentrations. The PCA (Figure 13) also indicated a strong positive correlation of LPO and ROS with O_3_ concentrations. Upon O_3_ exposure, the increased concentration of MDA contents and ROS, have been commonly reported in earlier studies [17,65,66,67,68,69]. Higher plants sustain an efficient enzymatic and non-enzymatic antioxidant pool to cope with O_3_-induced stress. In the present study, it was observed that enzymatic antioxidants decreased during the first sampling, while they increased during the second sampling in EO_1_ and EO_2_ ozone concentrations compared to AO. This suggests that the ROS scavenging mechanism was more efficient during the later stage of plant development. The variability in enzymatic responses at different developmental stages was also observed in cotton, where a lesser heterogeneity was recorded at the later sampling stage [70]. In the present study, the discrepancy of enzymatic response at different developmental stages in EO_1_ and EO_2_ plants can be well-correlated to the respective ROS contents. Whereas SOD catalyses the dismutation of O_2_^−0^ and H_2_O_2_, APX and CAT are responsible for scavenging H_2_O_2_ [18,71,72]. Non-significant variations in O_2_^−0^ accompanied by an increase in H_2_O_2_ contents demonstrate the rapid activity of SOD. The present study shows a reduction in ascorbate content at both sampling stages, which indicates its poor regeneration capacity. This observation can be linked to the inefficient upregulation of GR, pointing towards its more sensitive nature towards O_3_ stress [18]. The loading values of PCA for enzymatic antioxidants also supports this discussion (Figure 13). Notably, a higher increase in the enzyme activity was observed during the second sampling of the EO_1_ and EO_2_ treatments, as compared to AO treatments, suggesting an efficient stimulation of the defence machinery of lemongrass to combat the O_3_ stress at a later developmental stage. The incitement of antioxidant enzymes has also been reported in wheat [17,62], cotton [70], bean [65] and castor [66]. However, in this study, constitutive levels of enzymatic antioxidants were always higher at first sampling stage compared to the second sampling stage in all the treatments, suggesting their utilization of resources in other metabolic pathways during the later developmental stage. The response of DPPH, FRAP and ABTS also indicate the balance between the ROS generation and antioxidative scavenging in plants during EO_1_ and EO_2_ treatments. 

O_3_ stress in plants was visibly evident via the foliar injury in the leaves of lemongrass grown during EO_1_ and EO_2_ treatments. However, no visible injury was reported in plants at ambient O_3_. In the present study, the recorded FIP was 68.09 and 124.12% for EO_1_ and EO_2_ O_3_ treatments compared to AO. Leaf injury at elevated O_3_ concentrations is an important parameter as it results in the impairment of photosynthesis via the destruction of photosynthetic pigments. Carotenoids, proline and anthocyanins act as a quencher of ROS and function as antioxidants to protect chlorophyll molecules from photo-oxidative damage. The decline in these contents causes more damage to photosynthetic pigments. In the present study, the reduction in *Ps* was accompanied by a decline in *gs* in plants grown at elevated O_3_ as compared to AO, which is considered to be an adaptive feature to minimise the entry of O_3_ plants [73]. During stress, imbalanced osmotic solute disturbs the K^+^ flux from guard cells, resulting in the modification of guard cell turgidity and stomatal aperture [74]. A simultaneous decrease in *Ps* and *gs* in response to O_3_ stress has also been reported in many other studies [64,65,75,76]. The increase in *iCO_2_* during EO_1_ and EO_2_ treatments can be correlated with reduced *Ps* due to damage to photosynthetic machinery [76]. The impediment of photosynthesis is further strengthened by the reduction in Fv/Fm ratio at EO_1_ and EO_2_ treatments, as compared to AO, as recorded in the present study. Fv/Fm represents the photochemical efficiency of PS II, and its reduction under elevated O_3_ treatments can be considered an important indicator of O_3_-induced photoinhibition due to deactivation of PS II. The disrupted functioning of PS II further enhances ROS generation during light-dependent activities of photosynthesis, thus resulting in damaged thylakoids and the inactivation of the enzymes of the Calvin cycle [76,77]. The present study depicts a significant reduction in leaf number and area, which clearly invigorates the negative effect of O_3_ on plants’ assimilation capacity. Elevated O_3_ results in the production of thinner leaves and hampers the formation of new leaves, which explains the reduction in plant biomass [78]. The decline in plant biomass at elevated O_3_ conditions is widely cited in numerous studies [4,61,62]. 

**Figure 14:**
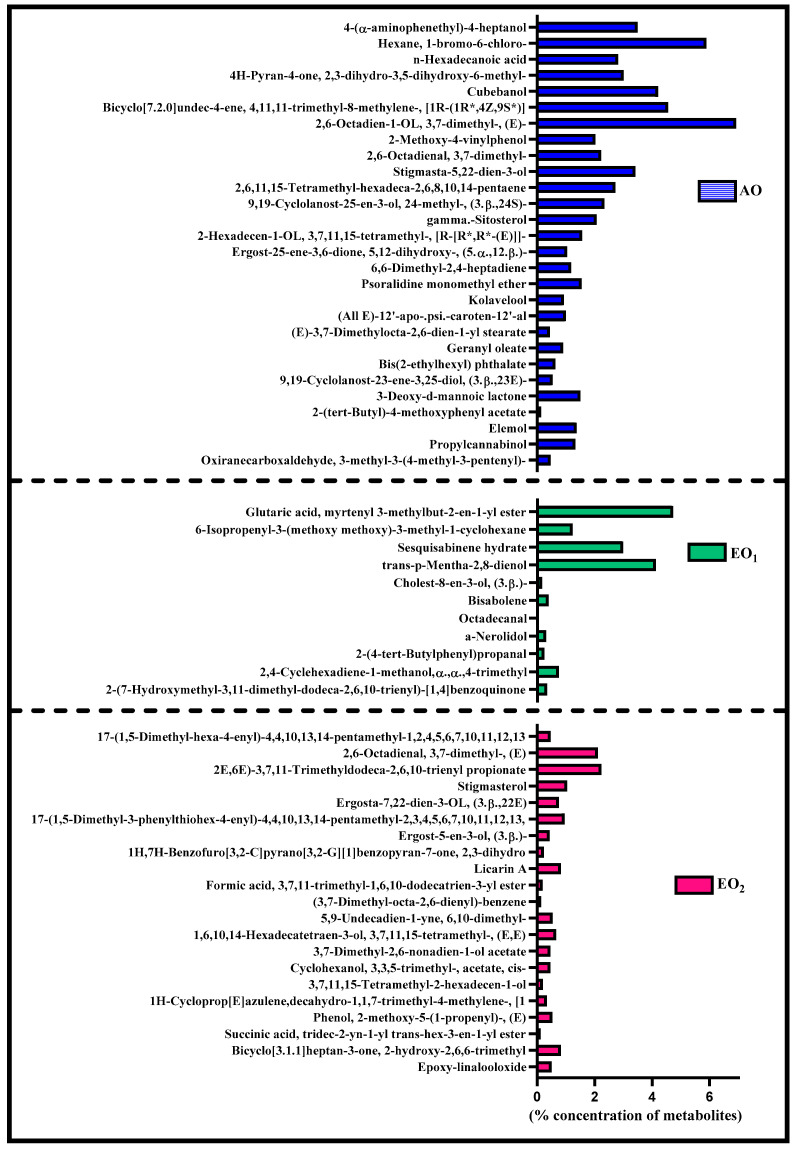
Number of uncommon * metabolites observed in leaf extract of lemongrass through GC–MS analysis at 110 days after transplantation (DAT) at ambient (AO) and elevated O_3_ (EO_1_ and EO_2_) treatments. * Metabolites present either in ambient (AO) or elevated O_3_ (EO_1_/EO_2_) treatments, specifically in the leaf extract.

**Figure 15:**
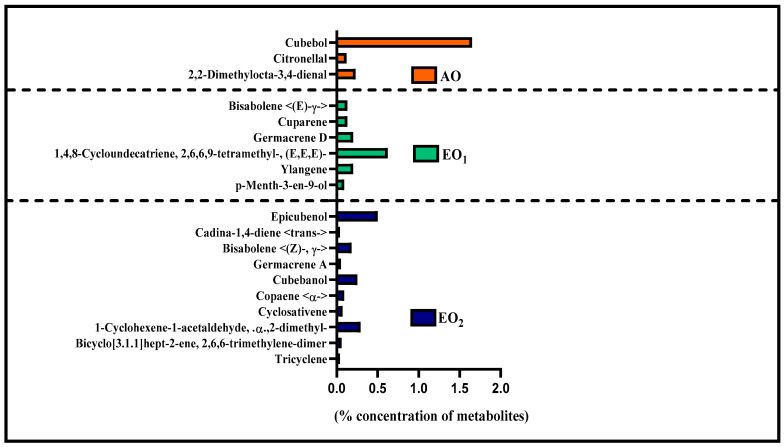
Number of uncommon metabolites observed in the essential oil of lemongrass through GC–MS analysis at 110 days after transplantation (DAT) at ambient (AO) and elevated O_3_ (EO_1_ and EO_2_) treatments. Metabolites present either in ambient (AO) or elevated O_3_ (EO_1_/EO_2_) treatments, specifically in the essential oil.

Secondary metabolite contents are an important constituent of the lemongrass, and are known to provide protection against O_3_-induced oxidative stress in plants [79,80]. Metabolites, such as phenolics, ascorbate and flavonoids, form an important defence line in plants, directly scavenging O_3_-related ROS [81]. Increments in the secondary metabolite contents under elevated O_3_ divert resources towards the shikimate–phenylpropanoid pathway. In the present study, the increase in flavonoid contents suggests that flavonoid biosynthesis genes were upregulated in plants under O_3_ stress conditions, thereby assisting the amelioration of ROS toxicity [64]. The GCMS data of the foliar extract and essential oil clearly demonstrate a huge heterogeneity in the composition and contents of the metabolites recorded at elevated O_3_ treatments, as compared to AO. In our study, the leaf extract showed 11 and 21 new compounds, while 6 and 10 new compounds were reported from essential oils during EO_1_ and EO_2_ treatments, respectively, as compared to the control (AO treatment) (Figure 14 and Figure 15). Although the foliar extract showed a reduction in the number of metabolites during EO treatments compared to AO, increments in the contents of many terpenes—such as camphorene (1.54 and 2.81%), geranyl acetate (6.11 and 12.16%), lanosterol (5.73 and 9.14%) and caryophyllene (1.65 and 2.01%)—were recorded (Figure 14; Supplementary Table S1). In addition, a few new compounds such as stearaldehyde (Octadecanal), steroids (stigmasterol, cholest-8-en-3-ol, ergost-5-en-3-ol), and polyphenolics (licarin A) were observed in the foliar extract of plants grown at elevated O_3_ (Figure 14; Supplementary Table S1). The increase in metabolite contents in plants during stress conditions were reported in earlier studies [25,60,82,83]. Caryophyllene, a sesquiterpene used in the treatment of osteroartheritis, diabetes and anxiety, has been reported to increase in *Sida cordifolia* [25], *Artemisia annua* [84] and *Zea mays* [85]. However, no change in the concentration of secondary metabolites was reported under stress conditions by Valkama et al. [86]. In the present study, the essential oils of O_3_-exposed plants also showed the appearance of new sesquiterpenes such as ylanglene, bisbolene, cuparene, epicubenol, and p-Menth-3-en-9-ol etc, which remarkably increased the medicinal properties of the essential oil (Figure 15). The modifications in the secondary metabolite profile of lemongrass upon O_3_ exposure can be explained by the upregulation of the genes associated with shikimate–phenylpropanoid pathway [87]. Being ubiquitous in plants, the compounds of the phenylpropanoid pathway have higher antioxidative properties, thereby ensuring an efficient scavenging of O_3_-generated ROS [88]. When plants are under abiotic stress such as O_3_, the shikimic–acid pathway is stimulated, which frequently results in the production of new metabolic compounds [89,90]. The appearance of new terpenoids in essential oils of plants treated with elevated O_3_ not only strengthens the plant’s defence, but also invigorates their medicinal value (Figure 15). Ylanglene shows anti-microbial and anti-diabetic activity, while bisbolene has anti-cancer properties. Cuparene is effective for regulating immunity and improving memory and epicubenol is helpful in the treatment of Alzheimer’s disease [91,92].

## 5. Conclusions

The elevated O_3_ inflicted significant negative responses in lemongrass, manifested in form of greater leaf injury, curtailed carbon fixation, and reduced plant biomass. The stimulation of enzymatic and non-enzymatic antioxidants was successful in scavenging O_3_-generated ROS to some extent. The magnitude of the response, however, was more at elevated O_3_ concentrations. The results of the study clearly depict that SOD was more responsive towards elevated O_3_ stress, whereas GR was not able to up regulate its activity, thereby affecting the AsA regeneration potential of the plant. It was further observed that the antioxidant system was more receptive during the later developmental stage, at both O_3_ concentrations. The reduction in biomass accompanied by the increase in the number of metabolites in leaf extract and essential oils suggest that plants are allocating more resources towards the phenylpropanoid pathway. O_3_ exposure not only resulted in and increased production of important metabolic compounds, it also induced the formation of new compounds, such as cuparene, bisbolene, epicubenol, in the essential oils, thus enhancing the medicinal properties and uses of lemongrass. Our results regarding the medicinal values of lemongrass, which will be prone to high O_3_ concentrations in the coming years, can be exploited by the pharmaceutical sectors. 

## Data Availability

The data presented in this study are available in article and supplementary material.

## Supplementary Materials

The following supporting information can be downloaded at: https://www.mdpi.com/article/10.3390/metabo13050597/s1, Table S1: Variation in the metabolic profile of leaf extract due to the EO exposure in *Cymbopogon flexuosus*; Table S2: Variation in the metabolic profile of essential oil due to the EO exposure in *Cymbopogon flexuosus* and Table S3: The IUPAC name and chemical structure of different compounds obtained by GC- MS in *Cymbopogon flexuosus*.
