# Supplementary material for: Role of Elevated Ozone on Development and Metabolite Contents of Lemongrass [Cymbopogon flexuosus (Steud.) (Wats.)]
Source: Metabolites. 2023 Apr 27;13(5):597. doi: 10.3390/metabo13050597 (PMC10223272; doi:10.3390/metabo13050597)
Supplement: Supplementary file 1 [file metabolites-13-00597-s001.zip › metabolites-2323917-supplementary.pdf]

Supplementary Table S1: Variation in the metabolic profile of leaf extract due to the EO exposure in *Cymbopogon flexuosus*

S. no.	Compounds	Retention peak area (%)		
		AO	EO_1	EO_2
1	Propylcannabinol	0.69	-	-
2	Elemol	0.71	-	-
3	2-(tert-Butyl)-4-methoxyphenyl acetate	0.07	-	-
4	3-Deoxy-d-mannoic lactone	0.78	-	-
5	Psoralidine monomethyl ether	0.8	-	-
6	6,6-dimethyl-2,4-heptadiene	0.61	-	-
7	2-hexadecen-1-ol, 3,7,11,15-tetramethyl-, [R- [R*, R*-(E)]	0.81	-	-
8	Sitosterol	1.07	-	-
9	2,6,11,15-Tetramethyl-hexadeca-2,6,8,10,14-pentaene	1.41	-	-
10	Stigmasta-5,22-dien-3-ol	1.77	-	-
11	2-methoxy-4-vinylphenol	1.05	-	-
12	2,6-octadien-1-ol, 3,7-dimethyl-, (E)-	3.59	-	-
13	Bicyclo [7.2.0] undec-4-ene, 4,11,11-trimethyl-8-methylene-, [1R-(1R*,4Z,9S*)]	2.36	-	-
14	Cubebanol	2.18	-	-
15	4H-Pyran-4-one, 2,3-dihydro-3,5-dihydroxy-6-methyl	1.56	-	-
16	Hexane, 1-bromo-6-chloro-	3.05	-	-
17	4-heptanol	1.81	-	-
18	2,4-cyclohexadiene-1-methanol, α, α.,4-trimethyl	-	0.7	-

19	trans-p-Mentha-2,8-dienol	-	3.87	-
20	Sesquisabinene hydrate	-	2.8	-
21	6-isopropenyl-3-(methoxymethoxy)-3-methyl-1-cyclohexene	-	1.15	-
22	Glutaric acid, myrtenyl 3-methylbut-2-en-1-yl ester	-	4.43	-
23	Cholest-8-en-3-ol	-	0.16	-
24	Octadecanal	-	0.05	-
25	Bicyclo[3.1.1]heptan-3-one, 2-hydroxy-2,6,6-trimethyl	-	-	0.83
26	17-(1,5-Dimethyl-3-phenylthiohex-4-enyl)-4,4,10,13,14-pentamethyl-2,3,4,5,6,7,10,11,12,13	-	-	0.96
27	Ergost-5-en-3-ol	-	-	0.76
28	Stigmasterol	-	-	1.04
29	2E,6E)-3,7,11-Trimethyldodeca-2,6,10-trienyl propionate	-	-	2.24
30	Citral	-	-	2.11
31	Licarin A	-	-	0.83
32	Linalool	0.55	-	0.43
33	m-Camphorene	0.79	1.54	2.81
34	Squalene	0.83	0.74	1.15
35	Palmitic acid	4.94	0.67	5.31
36	Lanosterol	4.33	5.73	9.14
37	Geranyl stearate	0.23	5.9	0.32
38	Geranyl linolenate	10.87	8.33	8.18
39	D:C-Friedo-B’: A'-neogammacer-9(11)-ene, 3-methoxy-, (3. β)-	3.37	5.97	0.9
40	Geranyl acetate	4.62	6.11	12.16
41	Geranyl linoleate	6.99	6.83	6.75

42	1,2,3,4,4a,5,6,8a-octahydro-7-methyl-4-methylene-1-(1-methylethyl)-, (1. α, / Neophytadiene	3.98	3.38	1.31
43	6-Methyl-4,6-bis(4-methylpent-3-en-1-yl) cyclohexa-1,3-dienecarbaldehyde	4.67	7.63	6.27
44	Vitamin E	1.67	1.88	2
45	9,19-Cyclolanost-25-en-3-ol, 24-methyl-, (3. β., 24S)-	2.21	2.58	-
46	Citronellyl linolenate	0.18	8.33	-
47	Cadinene < γ>	4.89	-	5.78
48	2,6,10,14-Hexadecatetraen-1-ol, 3,7,11,15-tetramethyl-, acetate, (E, E, E)-	0.64	-	1.11
49	Humulene < α>		5.68	0.42
50	β- Citral	-	1.54	11.71
51	Monopentyl Phthalate	-	1.04	0.31
52	Caryophyllene	-	1.65	2.01

Supplementary Table S2: Variation in the metabolic profile of essential oil due to the EO exposure in *Cymbopogon flexuosus*

**S. no.**	**Compounds**	**Retention peak area (%)**		
		**AO**	**EO_1**	**EO_2**
1	2,2-Dimethylocta-3,4-dienal	0.23	-	-
2	Citronellal	0.12	-	-
3	Cubebol	1.65	-	-
4	p-Menth-3-en-9-ol	-	0.09	-
5	α -Ylangene	-	0.2	-
6	1,4,8-cycloundecatriene, 2,6,6,9-tetramethyl-, (E, E, E)	-	0.62	-
7	Germacrene D	-	0.2	-

8	Cuparene	-	0.13	-
9	Bisabolene	-	0.13	-
10	Tricyclo [2.2.1.0(2,6)] heptane, 1,7,7-trimethyl	-	-	0.04
11	Bicyclo [3.1.1] hept-2-ene, 2,6,6-trimethyl	-	-	0.06
12	1-Cyclohexene-1-acetaldehyde, α.,2-dimethyl	-	-	0.29
13	Cyclosativene	-	-	0.07
14	Copaene < α>	-	-	0.09
15	Cubebanol	-	-	0.25
16	Germacrene A	-	-	0.05
17	Bisabolene <(Z)-, γ>	-	-	0.18
18	Cadina-1,4-diene <trans->	-	-	0.04
19	Epicubenol	-	-	0.5
20	Limonene	0.88	2.52	0.65
21	Geranyl linalool	1.25	1.24	1.75
22	Isogeranial	4.71	4.65	5.19
23	Cadinene < γ>	1.52	1.59	2.69
24	Neral	31.33	36.23	38.42
25	Geraniol	7.48	3.22	2.52
26	Citral	36.7	37.66	39.21
27	Geranyl acetate	4.7	2.19	6.06
28	Caryophyllene <(E)->	3.17	4.14	4.1
29	1,2,4-Metheno-1H-indene, octahydro-1,7a-dimethyl-5-(1-methylethyl)-, [1S-(1. α.,2. α	0.35	-	0.08
30	Camphene	0.14	-	0.39
31	Humulene < α>	0.44	-	0.61

32	1,6-cyclodecadiene, 1-methyl-5-methylene-8-(1-methylethyl)-, [s- (E, E**)**	0.21	-	0.63
33	Salvene <E->	0.12	0.08	-
34	Sulcatone	0.45	0.07	-
35	Cadinene < δ>	-	0.24	1.51

Supplementary Table S3: The IUPAC name and chemical structure of different compounds obtained by GC-MS in *Cymbopogon flexuosus*

S. no.	Compounds	IUPAC name	Chemical Structure	Molecular Formula
1	Propylcannabinol	dimethyl-prop-2-enyl-(6,6,9-trimethyl-3-propylbenzo[c]chromen-1-yl) oxysilane	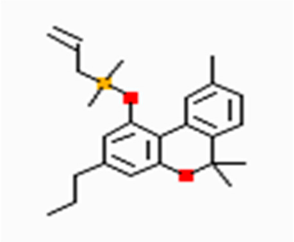	$C_{24}H_{32}O_2Si$
2	Elemol	Cyclohexane methanol, 4-ethenyl-α, α,4-trimethyl-3-(1-methylethenyl)-, [1R-(1α,3α,4β)]	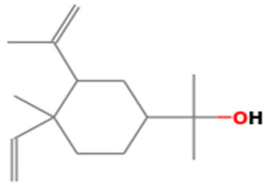	$C_{15}H_{26}O$
3	2-(tert-Butyl)-4-methoxyphenyl acetate	tert-butyl 2-(4-methoxy phenyl) acetate	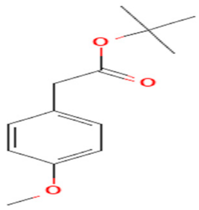	$C_{13}H_{18}O_3$
4	3-Deoxy-d-mannoic lactone	3,5-dihydroxy-6-(hydroxy methyl) oxan-2-one	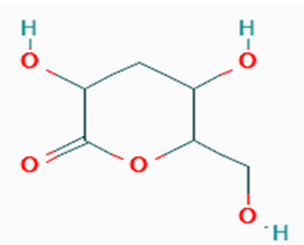	$C_6H_{10}O_5$
5				
6	Psoralidine monomethyl ether	Psoralidine monomethyl ether	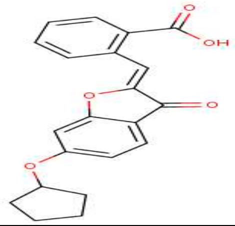	$C_{21}H_{18}O_5$
7	6,6-dimethyl-2,4-heptadiene	(2E,4Z)-6,6-dimethylhepta-2,4-diene	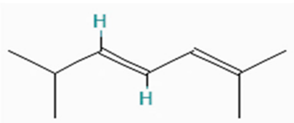	C_9H_{16}
8	2-hexadecen-1-ol, 3,7,11,15-tetramethyl-, [R-[R*, R*-(E)]	(E)-3,7,11,15-tetramethylhexadec-2-en-1-ol	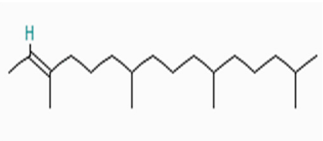	$C_{20}H_{40}O$

9	Sitosterol	(1*R*,3a*S*,3b*S*,7*S*,9a*R*,9b*S*,11a*R*)-1-[(2*R*,5*R*)-5-Ethyl-6-methylheptan-2-yl]-9a,11a-dimethyl 2,3,3a,3b,4,6,7,8,9,9a,9b,10,11,11a-tetradecahydro-1*H*-cyclopenta[*a*]phenanthren-7-ol	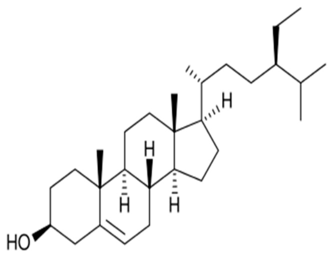	$C_{29}H_{50}O$
10	9,19-Cyclolanost-25-en-3-ol, 24-methyl-, (3. β.,24S)-	15-(5,6-dimethylhept-6-en-2-yl)-7,7,12,16-tetramethylpentacyclo $[9.7.0.0^{1,3}.0^{3,8}.0^{12,16}]$ octadecan-6-ol	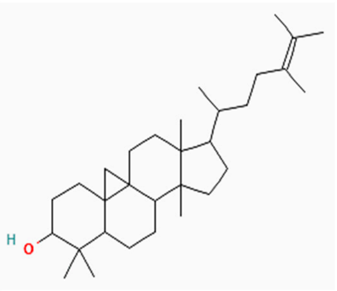	$C_{31}H_{52}O$
11	2,6,11,15-Tetramethyl-hexadeca 2,6,8,10,14-pentaene	(6E,8E,10E)-2,6,11,15-tetramethylhexadeca-2,6,8,10,14-pentaene	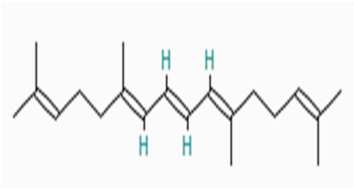	$C_{20}H_{32}$
12	Stigmasta-5,22-dien-3-ol	(3*S*)-17-[(*E*)-5-ethyl-6-methylhept-3-en-2-yl]-10,13-dimethyl-2,3,4,7,8,9,11,12,14,15,16,17-dodecahydro-1*H*-cyclopenta[a]phenanthren-3-ol	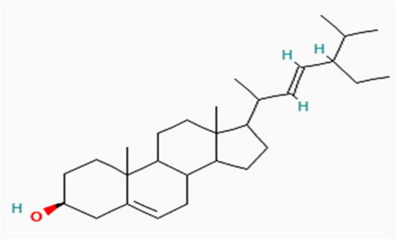	$C_{29}H_{48}O$
13	Citral	2,6-Octadienal, 3,7-dimethyl-, (E)-	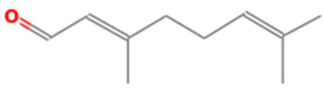	$C_{10}H_{16}O$
14	2-methoxy-4-vinylphenol	4-Ethenyl-2-methoxyphenol	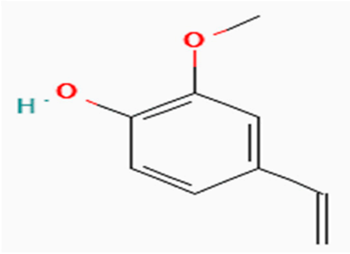	$C_9H_{10}O_2$

15	2,6-octadien-1-ol, 3,7-dimethyl-, (E)	3,7-dimethyl-octa-2,6-dien-1-ol	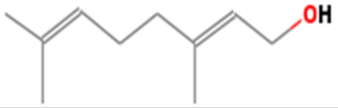	$C_{10}H_{18}O$
16	Caryophyllene	(1R,4E,9S)-4,11,11-trimethyl-8-methylidenebicyclo [7.2.0] undec-4-ene	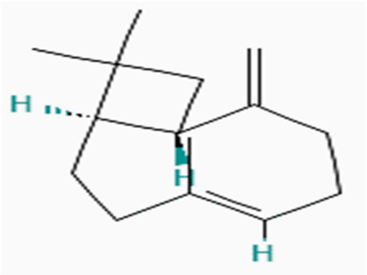	$C_{15}H_{24}$
17	Humulene < α>	(1*E*,4*E*,8*E*)-2,6,6,9-Tetramethylcycloundeca-1,4-8-triene	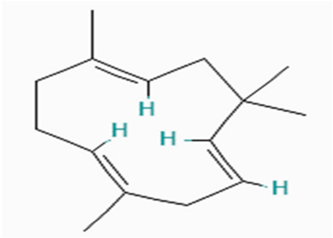	$C_{15}H_{24}$
18	Cubebanol	(1*R*,4*S*,5*R*,6*R*,7*S*,10*R*) -4,10-dimethyl-7-propan-2-yltricyclo [4.4.0.0^{1,5}] decan-4-ol	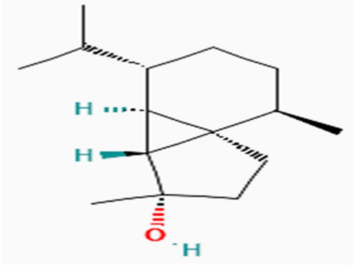	$C_{15}H_{26}O$
19	4H-Pyran-4-one, 2,3-dihydro-3,5-dihydroxy-6-methyl	3,5-dihydroxy-6-methyl-2,3-dihydropyran-4-one	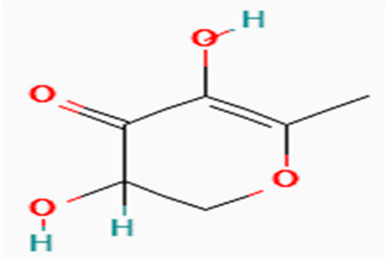	$C_6H_8O_4$
20	Hexane, 1-bromo-6-chloro	1-bromo-6-chlorohexane	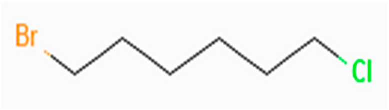	$C_6H_{12}BrCl$

21	4-heptanol	4-phenylheptan-4-ol	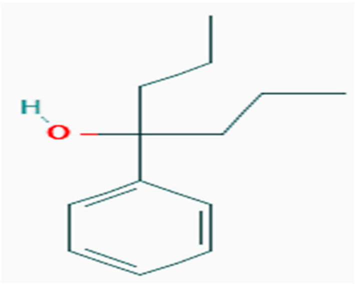	$C_{13}H_{20}O$
22	2,4 cyclohexadiene-1-methanol, α, α .,4-trimethyl	2,4-Cyclohexadiene-1- methanol, α, α 4-trimethyl-p-Mentha-1,5-dien-8-ol	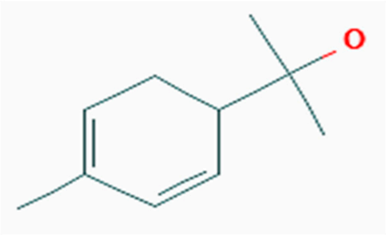	$C_{10}H_{16}O$
23	trans-p-Mentha-2,8-dienol	(3S,6R)-3-methyl-6-prop-1-en-2-ylcyclohexen-1-ol	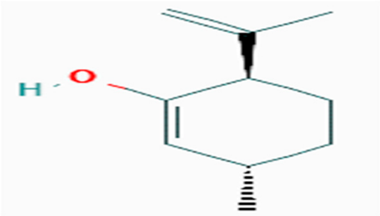	$C_{10}H_{16}O$
24	Sesquisabinene hydrate	2-methyl-5-(6-methylhept-5-en-2-yl) bicyclo [3.1.0] hexane-2-ol	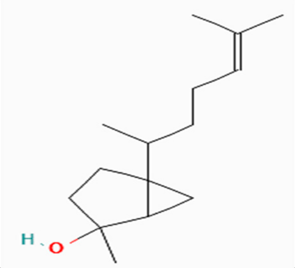	$C_{15}H_{26}O$
25	6-isopropenyl-3-(methoxy methoxy)-3-methyl-1-cyclohexene	3-(methoxy methoxy)-3-methyl-6-prop-1-en-2-ylcyclohexene	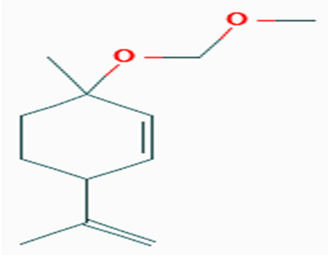	$C_{12}H_{20}O_2$
26	Glutaric acid, myrtenyl 3-methylbut-2-en-1-yl ester	5-O-[(2E)-3,7-dimethylocta-2,6-dienyl] 1-O-(3-methylbut-2-enyl) pentanedioate	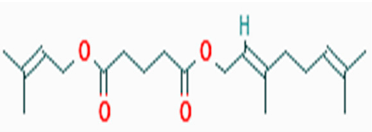	$C_{20}H_{32}O_4$

27	Cholest-8-en-3-ol	(9R,10S,13R,17R)-10,13-dimethyl-17-[(2R)-6-methylheptan-2-yl]-2,3,4,5,6,7,9,11,12,15,16,17-dodecahydro-1H-cyclopenta[a]phenanthren-3-ol	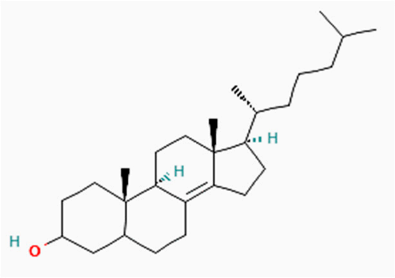	$C_{27}H_{46}O$
28	Octadecanal	Octadecanal	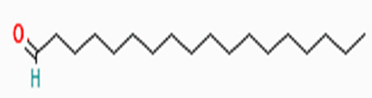	$C_{18}H_{36}O$
29	Bicyclo [3.1.1] heptan-3-one, 2-hydroxy-2,6,6-trimethyl	2-hydroxy-2,6,6-trimethyl bicyclo [3.1.1] heptan-3-one	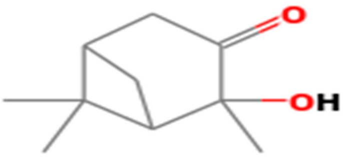	$C_{10}H_{16}O_2$
30	17-(1,5-Dimethyl-3-phenylthiohex-4-enyl)-4,4,10,13,14-pentamethyl-2,3,4,5,6,7,10,11,12,13	4-ethenyl-10,13-dimethyl-17-(6-methylheptan-2-yl)-2,3,4,5,6,7,8,9,11,12,14,15,16,17-tetradecahydro-1H-cyclopenta[a]phenanthren-3-ol	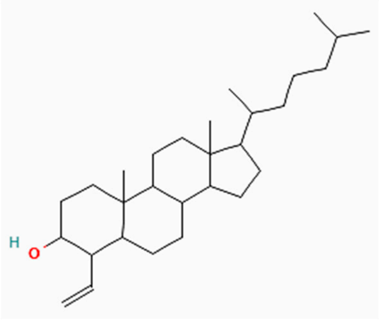	$C_{29}H_{50}O$
31	Ergost-5-en-3-ol	(8S,9S,10R,13R,14S,17R)-17-[(2R,5S)-5,6-dimethylheptan-2-yl]-10,13-dimethyl 2,3,4,7,8,9,11,12,14,15,16,17-dodecahydro-1H-cyclopenta[a]phenanthren-3-ol	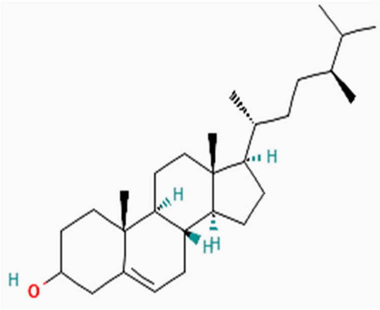	$C_{28}H_{48}O$
32	Stigmasterol	(3S,8S,9S,10R,13R,14S,17R)-17-[(E,2R,5S)-5-ethyl-6-methylhept-3-en-2-yl]-10,13-dimethyl-2,3,4,7,8,9,11,12,14,15,16,17-dodecahydro-1H-	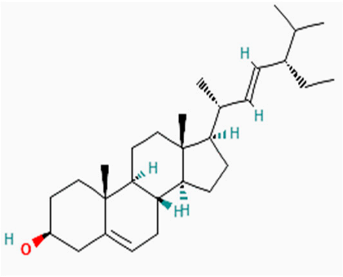	$C_{29}H_{48}O$

		cyclopenta[a]phenanthren-3-ol		
33	2E,6E)-3,7,11-trimethyldodeca-2,6,10-trienyl propionate	methyl 3,7,11-trimethyldodeca-2,6,10-trienoate	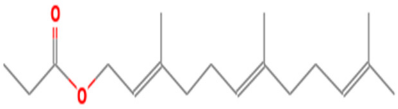	$C_{16}H_{26}O_2$
34	2,6-Octadienal, 3,7-dimethyl-, (E)	3,7-dimethylocta-2,6-dienal	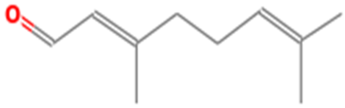	$C_{10}H_{16}O$
35	Licarin A	2-methoxy-4-(7-methoxy-3-methyl-5-prop-1-enyl-2,3-dihydro-1-benzofuran-2-yl) phenol	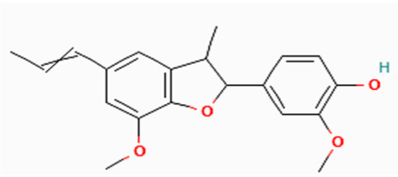	$C_{20}H_{22}O_4$
36	Geranyl linalool	3,7,11,15-tetramethylhexadeca-1,6,10,14-tetraen-3-ol	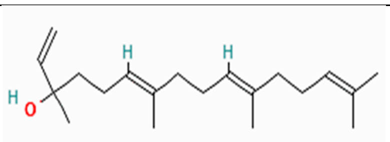	$C_{20}H_{34}O$
37	m- Camphorene	5-(6-methylhepta-1,5-dien-2-yl)-1-(4-methylpent-3-enyl) cyclohexene	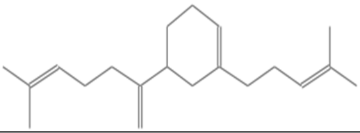	$C_{20}H_{32}$
38	Squalene	(6*E*,10*E*,14*E*,18*E*)-2,6,10,15,19,23-hexamethyltetracosa-2,6,10,14,18,22-hexaene	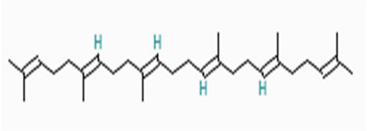	$C_{30}H_{50}$
39	Palmitic acid	[(2*Z*)-3,7-dimethylocta-2,6-dienyl] hexadecanoate	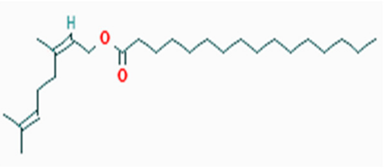	$C_{26}H_{48}O_2$
40	Lanosterol	(3*S*,5*R*,10*S*,13*R*,14*R*,17*R*)-4,4,10,13,14-pentamethyl-17-[(2*R*)-6-methylhept-5-en-2-yl]-2,3,5,6,7,11,12,15,16,17-decahydro-1*H*-cyclopenta[a]phenanthren-3-ol	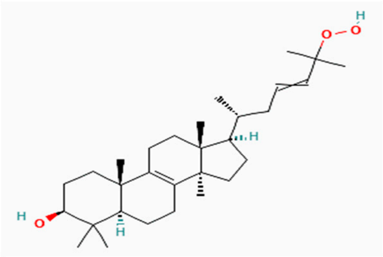	$C_{30}H_{50}O$

41	Geranyl stearate	[(2*E*)-3,7-dimethylocta-2,6-dienyl] octa decanoate	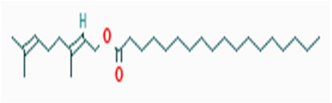	$\underline{C_{28}H_{52}O_2}$
42	Geranyl linolenate	[(2*E*)-3,7-dimethylocta-2,6-dienyl] (9*Z*,12*Z*,15*Z*)-octadeca-9,12,15-trienoate	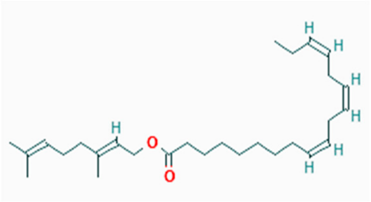	$\underline{C_{28}H_{46}O_2}$
43	D:C-Friedo-B': A'-neogammacer-9(11)-ene, 3-methoxy-, (3. β.)-	(9-acetyloxy-3a,5a,8,8,11a,13a-hexamethyl-3-propan-2-yl-1,2,3,4,5,5b,6,7,7a,9,10,11,13,13b-tetradecahydrocyclopenta[a]chrysen-10-yl) acetate	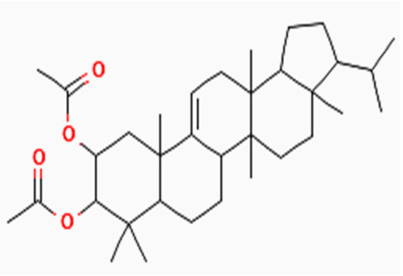	$\underline{C_{34}H_{54}O_4}$
44	Geranyl acetate	3,7-dimethylocta-2,6-dienyl acetate	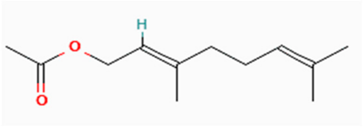	$C_{12}H_{20}O_2$
45	Geranyl linoleate	[(2*E*)-3,7-dimethylocta-2,6-dienyl] (9*Z*,12*Z*)-octadeca-9,12-dienoate	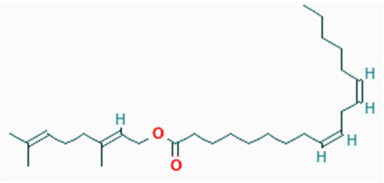	$\underline{C_{28}H_{48}O_2}$
46	1,2,3,4,4a,5,6,8a-octahydro-7-methyl-4-methylene-1-(1-methylethyl)-, (1. α, / Neophytadiene	1,2,3,4,4a,5,6,8a-octahydro-7-methyl-4-methylene-1-(1-methylethyl)-, (1. α,4aβ,8aα)	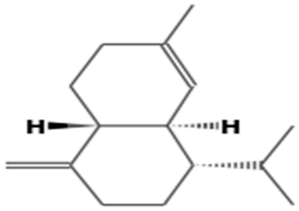	$C_{15}H_{24}$
47	6-Methyl-4,6-bis(4-methylpent-3-en-1-yl) cyclohexa-1,3-dienecarbaldehyde	6-Methyl-4,6-bis(4-methylpent-3-en-1-yl) cyclohexa-1,3-dienecarbaldehyde	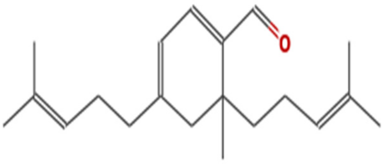	$C_{20}H_{30}O$

48	Vitamin E	(2R)-2,5,7,8-Tetramethyl-2-[(4R,8R)-4,8,12-trimethyltridecyl]-3,4-dihydro-2H-1-benzopyran-6-ol	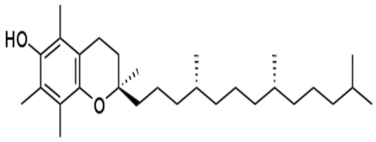	$C_{29}H_{50}O_2$
49	Citronellyl linolenate	3,7-dimethyloct-6-en-1-ol;(9*Z*,12*Z*,15*Z*)-octadeca-9,12,15-trienoic acid	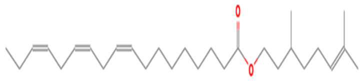	$C_{28}H_{48}O_2$
50	1,2,3,4,4a,5,6,8a-octahydro-7-methyl-4-methylene-1-(1-methylethyl)-, (1. α, Naphthalene	(3*S*,8*aS*)-5,8*a*-dimethyl-3-prop-1-en-2-yl-2,3,4,4*a*,7,8-hexahydro-1*H*-naphthalene	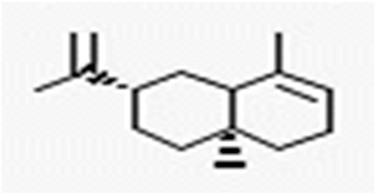	$C_{15}H_{24}$
51	2,6,10,14-Hexadecatetraen-1-ol, 3,7,11,15-tetramethyl-, acetate, (E, E, E)-	(2*Z*,6*E*,10*E*)-3,7,11,15-tetramethylhexadeca-2,6,10,14-tetraen-1-ol	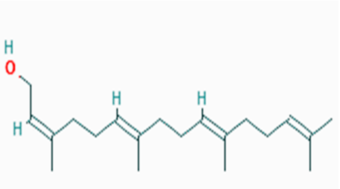	$C_{20}H_{34}O$
52	β-Citral	(3*E*)-3,7-dimethylocta-3,6-dienal	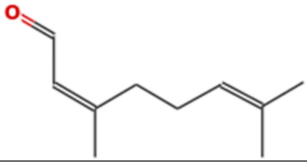	$C_{10}H_{16}O$
53	Monopentyl Phthalate	2-pentoxycarbonylbenzoic acid	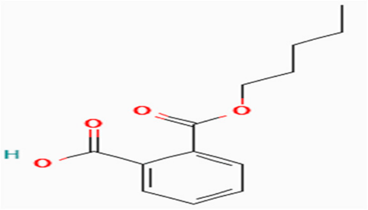	$C_{13}H_{16}O_4$
54	2,2-Dimethylocta-3,4-dienal	2,2-Dimethylocta-3,4-dienal	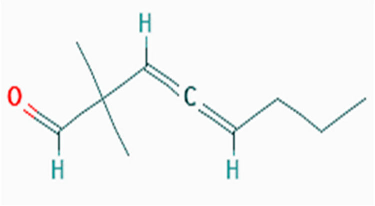	$C_{10}H_{16}O$
55	Citronellal	3,7-dimethyloct-6-enal	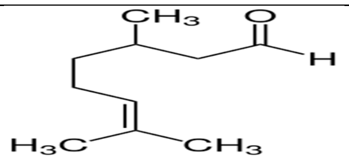	$C_{10}H_{18}O$

56	p-Menth-3-en-9-ol	2-(4-methylcyclohexen-1-yl) propan-1-ol	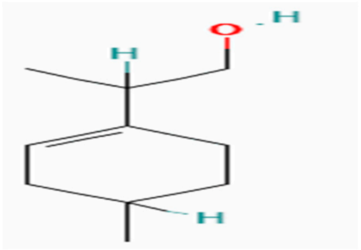	$C_{10}H_{18}O$
57	α - Ylangene	(1S,6R,7R,8S)-1,3-dimethyl-8-(propan-2-yl) tricyclo [4.4.0.0²,⁷]dec-3-ene	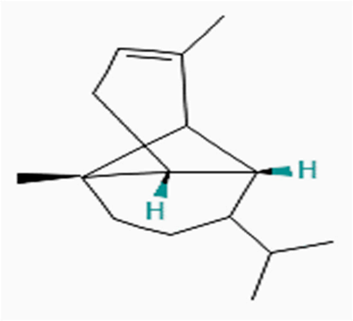	$C_{15}H_{24}$
58	Germacrene D	(S,1Z,6Z)-8-isopropyl-1-methyl-5-methylidenecyclodeca-1,6-diene	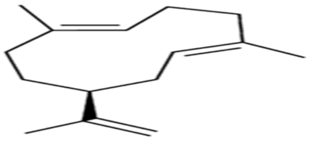	$C_{15}H_{24}$
59	Cuparene	1-methyl-4-[(1R)-1,2,2-trimethyl cyclopentyl] benzene	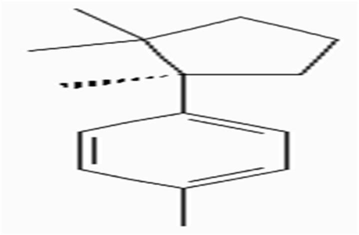	$C_{15}H_{22}$
60	Bisabolene	(4*S*)-1-methyl-4-(6-methylhepta-1,5-dien-2-yl) cyclohexene	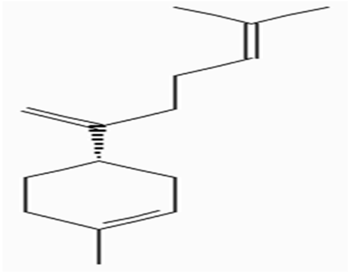	$C_{15}H_{24}$
61	Tricyclo [2.2.1.0(2,6)] heptane, 1,7,7-trimethyl	Tricyclo[2.2.1.0(2,6)] heptane, 1,7,7-trimethyl	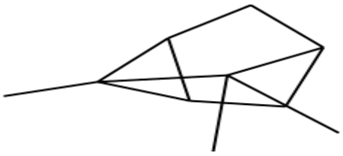	$C_{10}H_{16}$

62	Bicyclo [3.1.1] hept-2-ene, 2,6,6-trimethyl- dimer	2,6,6-trimethyl bicyclo [3.1.1] hept-2-ene	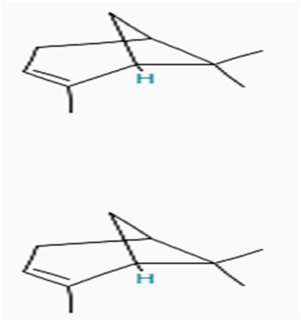	$C_{20}H_{32}$
63	1-Cyclohexene-1-acetaldehyde, α .,2-dimethyl	2-(2-methyl-5-prop-1-en-2-ylcyclohex-2-en-1-yl) propanal	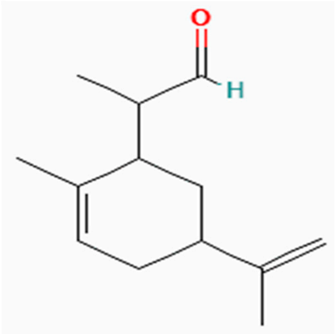	$C_{13}H_{20}O$
64	Cyclosativene	(1S,2S,3R,4S,6R,7R,8S)-1,2-dimethyl-8-propan-2- yl tetracyclo [4.4.0.0^{2,4}.0^{3,7}] decane	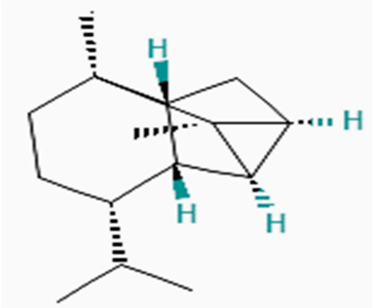	$C_{15}H_{24}$
65	Copaene <α>	(1R,2S,6S,7S,8S)-8-isopropyl-1,3-dimethyltricyclo [4.4.0.02,7] dec-3-ene	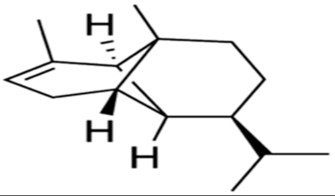	$C_{15}H_{24}$
66	Germacrene A	S,1Z,6Z)-8-isopropyl-1-methyl-5-methylidenecyclodeca-1,6-diene	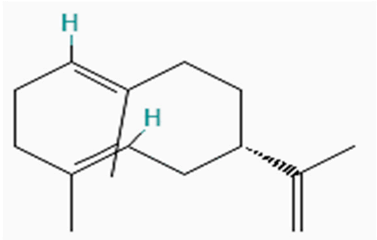	$C_{15}H_{24}$

67	Bisabolene <(Z), γ >	(4*Z*)-1-methyl-4-(6-methylhept-5-en-2-ylidene) cyclohexene	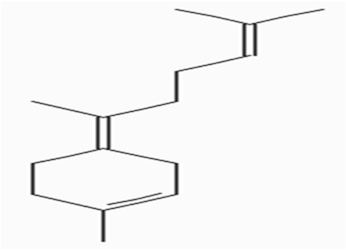	$C_{15}H_{24}$
68	Cadina-1,4-diene <trans->	(1S,4R)-1,6-dimethyl-4-propan-2-yl-1,2,3,4,4a,7-hexahydronaphthalene	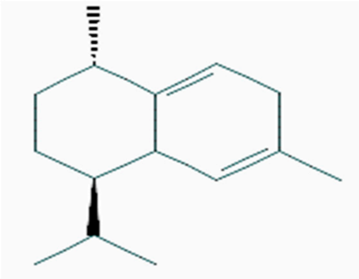	$C_{15}H_{24}$
69	Epicubenol	(1S,4R,4aS,8aR) -4,7-dimethyl-1-propan-2-yl-2,3,4,5,6,8a-hexahydro-1H-naphthalen-4a-ol	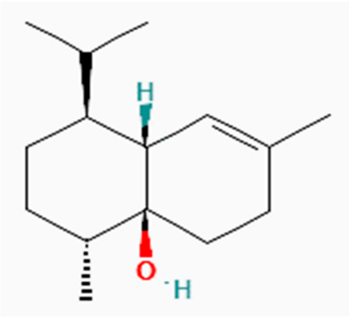	$C_{15}H_{26}O$
70	Limonene	1-Methyl-4-(prop-1-en-2-yl) cyclohex-1-ene	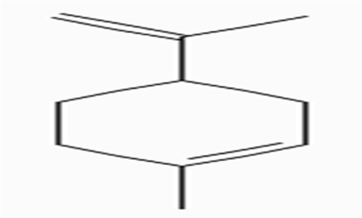	$C_{10}H_{16}$
71	Linalool	3,7-dimethyl-1,6-octadien-3-ol	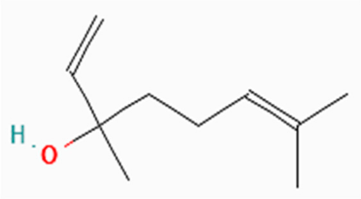	$C_{10}H_{18}O$

72	Isogeranial	(3*Z*)-3,7-dimethylocta-3,6-dien-1-ol	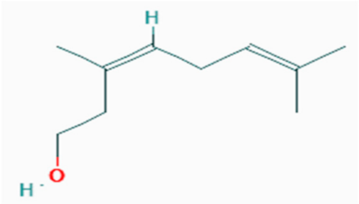	$C_{10}H_{18}O$
73	Cadinene < γ>	7-methyl-4-methylidene-1-propan-2-yl-2,3,4a,5,6,8a-hexahydro-1H-naphthalene	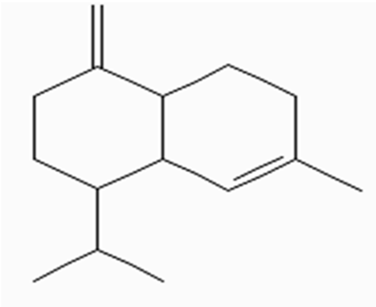	$C_{15}H_{24}$
74	Neral	(2Z)-3,7-dimethylocta-2,6-dienal	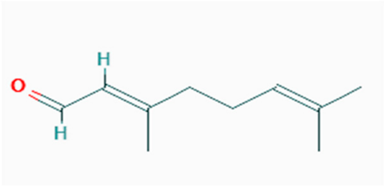	$C_{10}H_{16}O$
75	Geraniol	(2E)-3,7-Dimethylocta-2,6-dien-1-ol	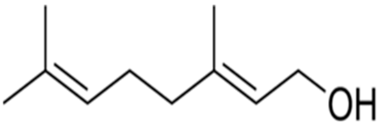	$C_{10}H_{18}O$
76	Camphene	3- [2-(3,3-dimethyl-2-bicyclo [2. 2. 1] heptanyl) ethenyl]-2,2-dimethyl bicyclo [2. 2.1] heptane	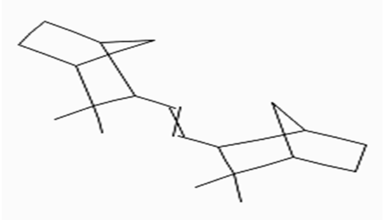	$C_{10}H_{16}$
77	Salvene <E>	(E)-6-methyl-5-methylidenehept-2-ene	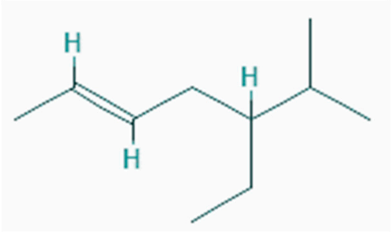	C_9H_{16}

78	Sulcatone	6-methylhept-5-en-2-one	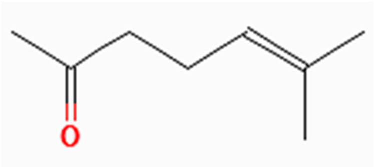	$C_8H_{14}O$
79	Cadinene < δ>	(1S,8aR)-4,7-dimethyl-1-propan-2-yl-1,2,3,5,6,8a-hexahydronaphthalene	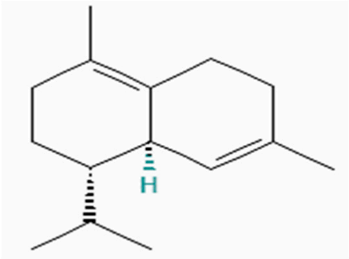	$C_{15}H_{24}$
